# Adherence to Injury Prevention Exercise Programmes in Amateur Adolescent and Adult Football: A Detailed Description of Programme Use from a Randomised Study

**DOI:** 10.1186/s40798-023-00608-1

**Published:** 2023-07-15

**Authors:** Hanna Lindblom, Markus Waldén, Martin Hägglund

**Affiliations:** 1 Unit of Physiotherapy, Division of Prevention, Rehabilitation and Community Medicine, Department of Health, Medicine and Caring Sciences, Linköping University, 581 83 Linköping, Sweden; 2 Sport Without Injury ProgrammE (SWIPE), Department of Health, Medicine and Caring Sciences, Linköping University, Linköping, Sweden; 3 Capio Ortho Center Skåne, Malmö, Sweden

**Keywords:** Implementation, Neuromuscular training, Fidelity, Soccer

## Abstract

**Background:**

Injury prevention exercise programmes (IPEPs) efficaciously reduce injuries. However, it is challenging to achieve sufficient adherence across a season. The main aim was to describe adherence to IPEPs in three groups of coaches and players partaking in a cluster randomised trial. Secondary aims were to describe perceptions of IPEPs, use of programme material, and overall preventive strategies.

**Methods:**

This is a sub-study analysing data from a three-armed randomised trial with teams randomised to use either a comprehensive IPEP (extended *Knee Control*) or an adductor strength programme, and non-randomised teams that already used a self-chosen IPEP regularly at study start (comparison group). Teams were instructed to use their respective intervention across the 2020 football season. Male and female, adolescent (≥ 14 years of age), and adult amateur players took part. Coaches and players responded to weekly and end-of-season questionnaires. Data were presented descriptively.

**Results:**

Weekly player data were reported by 502 players (weekly response rate 65%), 289 (58%) responded to end-of-season questionnaires. Teams in the extended *Knee Control* and comparison group used their respective IPEP in 483/529 (91%) and 585/641 (91%) of training sessions, and teams in the adductor group in in 199/315 (63%) sessions. Regarding utilisation fidelity, 42–52% of players in each group used 1–10 repetitions per exercise. Seven out of 17 teams in the adductor group had progressed as recommended in terms of number of repetitions. Two teams (10%) in the extended *Knee Control* group, and 7/24 of comparison teams used the same exercises across the season. Coaches accessed the IPEPs by different means (printed material, films, workshops, etc.), but half did not use the provided digital material. The players appreciated that the IPEPs could reduce injury risk and that they improved their exercise performance, but disliked that they had less time for football and that the exercises were boring. Forty-nine players had experienced pain during IPEP performance.

**Conclusions:**

Adherence with the IPEPs was generally high. To meet different coach needs, programme material should be available via different digital and printed resources. The number of players reporting pain during IPEP performance is a noteworthy finding.

***Trial registration*:**

Clinical Trials gov identifier: NCT04272047. Registered February 17, 2020. https://clinicaltrials.gov/ct2/show/NCT04272047.

**Supplementary Information:**

The online version contains supplementary material available at 10.1186/s40798-023-00608-1.

## Key Points

- Overall adherence with the injury prevention exercise programmes was high in all three groups (the extended *Knee Control*, adductor and comparison groups).
- Programme material should be spread by different means (social media, webpage, printed material, workshops) since a one-size-fits-all solution will not accommodate the needs of all coaches.
- Pain during preventive training is seen in one fifth of players and warrants further investigation, as this may be a barrier to injury prevention exercise programme use.

## Introduction

Multi-component injury prevention exercise programmes (IPEPs) for lower extremity injuries, such as the *Knee Control* programme, and the *FIFA 11*+ efficaciously prevent injuries [1, 2]. Short and targeted IPEPs, such as the *Adductor Strengthening Programme* [3] and the *Nordic Hamstring Exercise* [4, 5] also reduce injury rates. When comparing *Knee Control* effectiveness in a real-world context [6] versus the efficacy shown in a randomised controlled trial [1], effectiveness is considerably lower. Effectiveness is affected by implementation-related factors, such as programme adoption, implementation, adherence, and maintenance [7].

Adherence is a multidimensional implementation outcome describing how an intervention has been used [8]. A sufficient training frequency is vital for maximum preventive effect [9–12]. However, coaches often modify programme content and/or dosage [8, 13, 14]. Short and targeted IPEPs are less time-consuming than multiple component programmes, but they too suffer from low adoption and adherence [15–18]. Informed by previous studies, an extended *Knee Control* programme was developed from the original *Knee Control* programme, with more exercise options to facilitate tailoring of the programme and to increase programme fit, thereby aiming to improve feasibility and adherence [19]. Our main randomised trial showed that extended *Knee Control* was efficacious in reducing hamstring, knee, and ankle (combined) injury rates [19] and the present sub-study contains an analysis of additional data collected within the trial.

The main aim was to describe adherence to IPEPs in three groups of coaches and players partaking in a cluster randomised trial. Secondary aims were to describe perceptions of IPEPs, use of programme material, and overall preventive strategies.

## Methods

This study is based on questionnaire data collected in a three-armed cluster randomised trial in one regional football district in Sweden in 2020 [19]. A detailed flow diagram of the inclusion of players can be found in the main study [19] and in this sub-analysis follow-up data are presented for those who took part in the end-of-season questionnaires. The total season ranged 27–29 weeks for included teams (March to October), with a pre-season over 10 weeks and competitive season over 17–19 weeks. Teams were randomised either to the more extensive general IPEP “extended *Knee Control*” (*n* = 17, 7 male, 10 female) or an “adductor group” (*n* = 12, 5 male, 7 female) using a short single-exercise adductor strength programme. Teams that at baseline had already adopted and used injury prevention exercises on a regular basis took part in a “comparison group” (non-randomised, *n* = 17, 8 male, 9 female, one male team dropped out). Since the comparison group already used injury prevention exercises, whereas the other two groups did not, the comparison group could not be randomised. The comparison group was believed to represent a “best-case real-world injury prevention example” of IPEP use. The study was designed primarily to compare the programmes regarding preventive efficacy. The two IPEPs in the randomised groups had substantial differences regarding their overall content, extent, and recommendations for dosage and progression. Therefore, we describe adherence with each respective programme in relation to its specific recommendations rather than comparing adherence between the programmes.

The manuscript has been checked against the STROBE checklist [20].

### Important Modifications Due to the COVID-19 Pandemic

The study was carried out during the first year of the COVID-19 pandemic. Due to the pandemic, the start of the competitive season was postponed, and the pre-season was extended. Football training was never cancelled and injury prevention training could continue throughout the season. On April 1st, 2020, the Swedish Public Health Authority discouraged close contact between people. Due to this recommendation, we provided individual exercise options, as described under “Interventions”. Considering the higher injury rates during matches, the shorter competitive season may potentially have resulted in lower injury rates than during a normal season.

### Participants

The teams included in the study participated in a male or female adolescent or adult league 2020 series in one football district, and had at least two scheduled training sessions per week. Teams in the randomised arms had not engaged in regular prevention training during the previous year, while teams in the non-randomised arm had used an IPEP regularly at least once per week during the previous year, and planned to do so also in the 2020 season. Players ≥ 14 years of ages from these teams were eligible.

Coaches for all potentially eligible teams were approached via e-mail and telephone and asked about inclusion in the study, and received oral and written information about the study. Coaches then forwarded oral and written information to the players of their team. Response to the questionnaires was taken as consent to participate.

### Interventions

Workshops, where the respective intervention was introduced and exercises were practised, were offered to one coach and two to three players per team in the two randomised arms. After this introduction workshop the coaches were expected to lead the preventive training in their team. Ten teams had not taken part in the workshops by the time the pandemic began, and group gatherings were discouraged by the Swedish Public Health Authority. Nine of these teams were visited by a physiotherapist during their training in the beginning of the season and introduced to their intervention following the same set-up as during group workshops. The final team was introduced to the adductor strength programme via telephone by a study physiotherapist responsible for this intervention. Since few teams had access to medical support, the workshops specifically targeted coaches and players. In both intervention groups, programme material was distributed as (a) a printed folder with pictures and instructions for all exercises, (b) the same folder in digital format (PDF) (Additional files 1, 2), and (c) a link to a designated website containing films and instructions. The teams were recommended to start with their intervention immediately after taking part in the workshop, or after the physiotherapist site visit, and use the programme throughout the season (March–October 2020). One month after training began, we had telephone check-ups with coaches for all teams.

Teams in the comparison group were asked to continue with their ongoing training as usual and did not receive any programme material or training instructions within the study.

#### Extended Knee Control

Extended *Knee Control* was a 15–20-min IPEP with 5 min of running warm-up and 10–15 min of strengthening and neuromuscular control exercises (six main exercises with ten different exercise variations each, 60 exercise options in total). The teams were free to decide when to use the exercises during football practice.

The following recommendations were given:

- Use the programme at every training session
- Choose one exercise option for each main exercise and use for 30–60 s and for 2 sets
- Vary and progress training over time

#### The Adductor Strength Programme

The adductor group used exercises targeting hip and groin muscles (focus on adductors) with a similar set-up and dosage as Harøy et al. [3], but with two extra exercises added for teams who wanted to avoid close contact due to the pandemic. The programme contained dynamic adductor exercises on three different levels with progressively higher loads, and two isometric exercises. The programme took about 5 min to complete.

The following recommendations were given:

- Start with the most advanced level (Copenhagen adduction long lever)
- Use easier levels (Copenhagen adduction short lever, or side-lying adduction) if the players are unable to perform the exercise with correct technique or if players experience pain > 3 on a 0–10 numerical rating scale
- Number of repetitions per session and training frequency were progressively increased from 3–5 repetitions 2 times/week in week 1, to 3–5 repetitions and 3 times/week in week 2, 7–10 repetitions and 3 times/week in week 3–4, to 12–15 and 3 times/week in week 5–6 and 12–15 repetitions and 2 times/week in week 7–8 during pre-season
- Use the programme once per week and 12–15 repetitions during the competitive season
- Use exercises without close contact if preferred, adductor squeeze supine with straight legs and ball between feet, and adductor squeeze with bent legs and ball between knees [21], with 5 maximal isometric contractions for 10 s each.

### Data Collection

Data on weekly use of IPEPs and cumulative utilisation were reported in weekly questionnaires by one coach per team, and by all players during the season. The first weekly questionnaire was sent out approximately 4 weeks after the workshops. End-of-season questionnaires were distributed to coaches and players in October–November 2020. The questionnaires covered detailed descriptions of the participants’ use of their respective IPEP, different measures used to prevent injuries in addition to the IPEPs to get an overall picture of injury prevention practices, and perceptions of IPEP use since we believe the perceptions may associate with IPEP adherence. The questionnaires were custom-made to cover different aspects of IPEP use and pilot tested on physiotherapists and coaches prior to the study. Since the IPEPs differed in extent and recommendations, the end-of-season questionnaires were customised to each group, rendering 3 versions for players and 3 versions for coaches. Differences between the questionnaires were minor, such as the following question for players “What do you like with extended *Knee Control*” that was worded “What do you like with the adductor programme” and “What do you like with the injury prevention training” and with slight differences in response alternatives based on the different training recommendations.

All questionnaires were distributed via online software (esMakerNX3 V 3.0) through links sent out via e-mail and/or text messages. Two reminders were sent out via the online system and we also sent printed questionnaires to participants when a postal address was available. Players who ended their football season prematurely, e.g. due to injury, also received the end-of-season questionnaire.

### Analysis

Results were presented descriptively, and missing data were not imputed. Weekly use of the respective programme is presented for coaches and players. Adherence was treated as a multidimensional construct [22], and described in terms of cumulative utilisation (number and proportion of IPEP training sessions in relation to all training sessions), utilisation frequency (number of times per week using the respective IPEP), duration fidelity (number of minutes devoted to IPEP use each session), and utilisation fidelity (number of repetitions per exercise, and how, where, and when the IPEP was used). Data for cumulative utilisation and utilisation frequency were collected from the weekly questionnaires and for duration and utilisation fidelity from the end-of-season questionnaires. We also treated adherence as a multilevel construct and report use of the IPEPs separately for coaches/teams and for players. Use of programme material is presented for the two randomised groups separately, with some questions rated on a 1–7 Likert scale, where 1 represented the least and 7 the most favourable response. For extended *Knee Control*, we analysed use of all six main exercises for each team. Each exercise was assigned 0 to 4 points depending on how often it had been used during the season; 0 never, 1 seldom, 2 sometimes, 3 often, 4 always. In total maximum 24 points could be awarded (6 exercises × 4 points) if all six main exercises were used in every training session.

No sample size calculation was made for this sub-study, which included the total sample of responding players and coaches from the randomised trial. IBM SPSS Statistics for Windows (Version 27.0. Armonk, New York) was used for all analyses.

## Results

Sixty-one coaches (mean age 44.8 ± 8.2, 50 male) out of 81 coaches (75%), representing 46 teams, and 289 players (mean age 20.2 ± 5.6, 60 male), representing 44 teams, responded to the end-of-season questionnaire (Table 1). In the main study, 502 players took part and provided weekly data for the sub-study (weekly response rate 65%), and 289 (58%) of these players responded to the end-of-season questionnaire.

**Table 1:** Descriptive information about participants, dosage, and progression of the respective injury prevention programme

	Extended *Knee Control* group		Adductor group		Comparison group	
	*n* = 20 coaches	*n* = 120 players	*n* = 17 coaches	*n* = 64 players	*n* = 24 coaches	*n* = 105 players
Age (years), mean ± SD*	47.6 ± 9.0	19.3 ± 4.3	45.9 ± 6.7	21.4 ± 6.0	41.6 ± 7.9	20.5 ± 6.6
Sex, *n* male/female (% male)	19/1 (95.0)	28/92 (23.3)	12/5 (70.6)	10/54 (15.6)	19/5 (79.2)	22/83 (21.0)
**Cumulative utilisation**						
Number of IPEP sessions in total, *n*	483	4363	199	1644	585	3239
Training sessions in total, *n*	529	5451	315	2616	641	3836
Proportion of training sessions with IPEP (%)	91.3	80.0	63.2	62.8	91.3	84.4
**Utilisation frequency**						
Full season, times/week ± SD	2.2 ± 0.3	1.6 ± 1.2	1.3 ± 0.5	1.0 ± 0.9	2.1 ± 0.4	1.4 ± 1.2
Pre-season, times/week ± SD	2.3 ± 0.2	N/A	1.7 ± 0.2	N/A	1.9 ± 0.3	N/A
Competitive season, times/week ± SD	2.1 ± 0.3	N/A	0.7 ± 0.3	N/A	2.1 ± 0.4	N/A
**Duration fidelity**^†^						
Minutes spent on IPEP each session, mean ± SD	15.6 ± 4.3	16.6 ± 7.0	10.1 ± 4.6	7.1 ± 3.3	15.9 ± 7.2^‡^	14.6 ± 8.8
**Utilisation fidelity**^†^						
Mean number of repetitions per exercise						
1–5	6 (30.0)	12 (10.0)	N/A	9 (14.3)	5 (20.8)	22 (21.0)
6–10	6 (30.0)	47 (39.2)	N/A	24 (38.1)	6 (25.0)	22 (21.0)
11–15	6 (30.0)	40 (33.3)	N/A	23 (36.5)	5 (20.8)	27 (25.7)
16–20	1 (5.0)	11 (9.2)	N/A	4 (6.3)	2 (8.3)	16 (15.2)
> 20	1 (5.0)	10 (8.3)	N/A	3 (4.8)	6 (25.0)	18 (17.1)
‘How did you use your programme at training?’						
Same exercise(s) throughout the season	2 (10.0)	25 (20.8)	6 (35.3)	35 (55.6)	7 (29.2)	N/A
Different exercises for variation	18 (90.0)	93 (77.5)	5 (29.4)	14 (22.2)	13 (54.2)	N/A
More advanced exercises over time	6 (30.0)	18 (15.0)	8 (47.1)	15 (23.8)	4 (16.7)	N/A
Individual adaptation	1 (5.0)	7 (5.8)	8 (47.1)	4 (6.3)	3 (12.5)	N/A
Exercises without close contact due to COVID-19	2 (10.0)	N/A	0 (0.0)	N/A	3 (12.5)	N/A
Replaced or added exercises	5 (25.0)	N/A	2 (12.5)	N/A	N/A	N/A
‘When did you do the exercises?’						
Before football training, % of weeks	6.6	N/A	0.0	N/A	7.8	N/A
As part of the warm-up before football training, % of weeks	75.6	N/A	61.6	N/A	70.2	N/A
Imbedded in the football training, % of weeks	12.8	N/A	27.7	N/A	15.0	N/A
After football training, % of weeks	2.3	N/A	0.0	N/A	4.1	N/A
As part of warm-up before football match, % of weeks	7.8	N/A	0.0	N/A	14.7	N/A
Other, % of weeks	15.5	N/A	13.6	N/A	15.0	N/A
‘Where did you do the exercises?’						
At football training	N/A	119 (99.2)	N/A	61 (96.8)	N/A	102 (97.1)
At football matches	N/A	46 (38.3)	N/A	0 (0.0)	N/A	36 (34.3)
At home	N/A	14 (11.7)	N/A	7 (11.1)	N/A	42 (40.0)

Values are *n* (%) unless otherwise stated. N/A (not applicable) represent instances where this specific group of coaches or players did not receive the question

Teams using extended *Knee Control* were recommended to use the programme for 15–20 min each training session, each exercise for 30–60 s and in two sets. They were also recommended to progress training over time with more advanced exercises. Teams using the adductor programme were recommended to use the most advanced exercise (Copenhagen adduction long lever) and progress from 3–5 repetitions to 12–15 during pre-season. They were recommended to follow a structured progression plan (repetitions and set) during pre-season, and thereafter to use maintenance training once per week during the competitive season. Alternative exercises (Copenhagen adduction short lever, side-lying adduction, adductor squeeze) were recommended for players with pain, those unable to do the Copenhagen adduction exercise long lever or those wishing to avoid close contact due to COVID-19

COVID-19, Coronavirus Disease 2019; N/A, not applicable; SD, standard deviation. Utilisation frequency for teams is represented with weeks when the team had cancelled its training sessions (for example due to Easter or Summer break) excluded, whereas player utilisation frequency is calculated with all weeks included for which players have responded. Regarding when the exercises were used, coaches could respond with multiple alternatives

*Missing data for 1 player in adductor group, 2 players in comparison group, 4 coaches in the extended Knee Control group, 3 in the adductor group and 6 in the comparison group

^†^Missing data for one player in the adductor group

^‡^One outlier excluded who probably had misinterpreted the question

### Use of the Preventive Programmes Across the Season

**Fig. 1:**
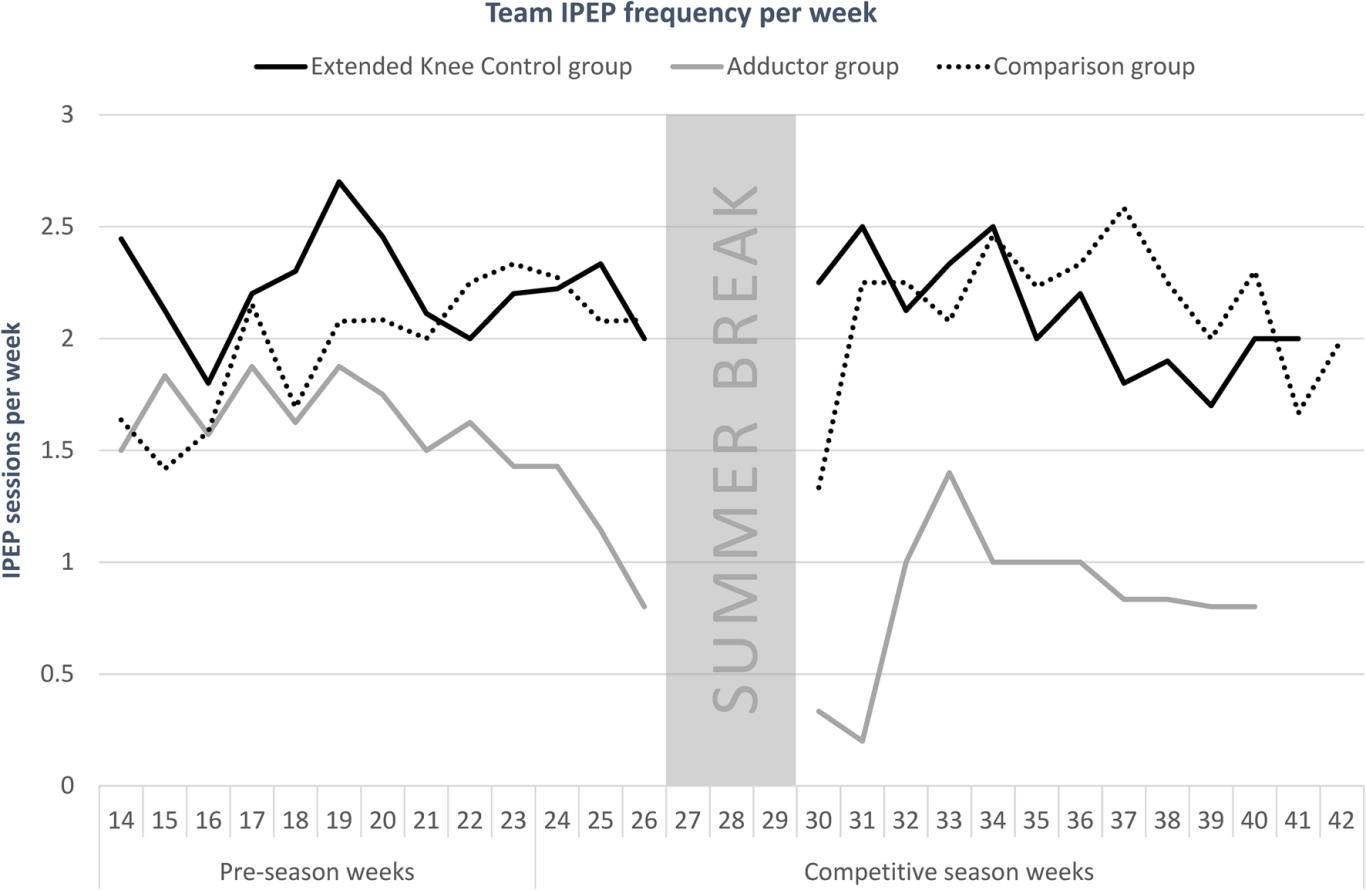
Average use of injury prevention exercise programmes, as reported by coaches on a weekly basis. *IPEP* injury prevention exercise programme

Teams in the extended *Knee Control* group and the comparison group used their respective IPEP in 91% of all training sessions. The adductor programme, with its varying recommendations with 2–3 sessions/week during pre-season and 1 session/week during competitive season, was used in a total of 63% of training sessions (Table 1). Weekly use of the IPEPs in the extended *Knee Control* group and the comparison group was similar throughout the season, however it was lower during the competitive season in the adductor group, in line with programme recommendations (Fig. 1).

Regarding utilisation fidelity, 49%, 52%, and 42% of players in the extended *Knee Control* group and the adductor and comparison groups, used only 1–10 repetitions per exercise, respectively (Table 1). The majority of coaches in all three groups indicated that they used their IPEP together with the warm-up, but up to one fourth of coaches (in the adductor group) had embedded the exercises in football training. Ten percent of coaches in the extended *Knee Control* group (21% of players), 35% of coaches in the adductor group (56% of players), and 29% of coaches in the comparison group used the same exercises throughout the season. The majority of coaches and players in the extended *Knee Control* group had used different exercises for variation. Only 5 and 2 coaches in the extended *Knee Control* group and the adductor group, respectively, reported that they had modified the programme, and 4 (2 in the extended *Knee Control* group, 2 in the adductor group) did this due to pain experienced by individual players.

In the extended *Knee Control* group, all six main exercises were used “sometimes” to “always” by most players and coaches (Table 2). In addition to analysing each exercise separately, we analysed use for each team, where each exercise was assigned between 0 (never used) to 4 (always used) points. The 16 responding teams received between 12 and 24 points (maximum 24 points); nine teams received > 20 points, indicating that they had used most of the six exercises “almost always”, three teams scored 18 points, and four scored less than 16 points.

**Table 2:** Description of how often each coach (*n* = 20) and player (*n* = 120) in the extended *Knee Control* group indicated that they had used the respective exercise component

	Never		Seldom		Sometimes		Often		Always	
	Coaches	Players	Coaches	Players	Coaches	Players	Coaches	Players	Coaches	Players
Running warm-up	1 (5.0)	1 (0.8)	3 (15.0)	4 (3.3)	2 (10.0)	12 (10.0)	5 (25.0)	30 (25.0)	9 (45.0)	73 (60.8)
One-legged knee squats	0 (0.0)	2 (1.7)	0 (0.0)	8 (6.7)	4 (20.0)	29 (24.2)	8 (40.0)	47 (39.2)	8 (40.0)	34 (28.3)
Hamstring strengthening	0 (0.0)	2 (1.7)	0 (0.0)	13 (10.8)	4 (20.0)	36 (30.0)	12 (60.0)	47 (39.2)	4 (20.0)	22 (18.3)
Two-legged knee squats	0 (0.0)	0 (0.0)	1 (5.0)	3 (2.5)	2 (10.0)	28 (23.3)	8 (40.0)	49 (40.8)	9 (45.0)	40 (33.3)
Core strengthening	0 (0.0)	2 (1.7)	0 (0.0)	12 (10.0)	5 (25.0)	34 (28.3)	9 (45.0)	42 (35.0)	6 (30.0)	30 (25.0)
Lunges	0 (0.0)	0 (0.0)	0 (0.0)	5 (4.2)	3 (15.0)	29 (24.2)	9 (45.0)	53 (44.2)	8 (40.0)	33 (27.5)
Jump/landing technique	0 (0.0)	1 (0.8)	1 (5.0)	11 (9.2)	2 (10.0)	31 (25.8)	6 (30.0)	40 (33.3)	11 (55.0)	37 (30.8)
Elastic tubes	2 (10.0)	35 (29.2)	9 (45.0)	32 (26.7)	4 (20.0)	33 (27.5)	4 (20.0)	18 (15.0)	1 (5.0)	2 (1.7)

Numbers represent *n* coaches/players and (percent)

In the adductor group, the two Copenhagen adduction exercises (short and long lever) were used by most coaches (82%), and the additional adductor squeeze exercises were used by the fewest (35–47%) number of coaches. Many players had used the Copenhagen adduction exercises (58–63%), but even more (72%) had used side-lying adduction (Table 3). During pre-season, the majority of coaches had used the lower prescribed training volume, with 3–5 repetitions and 2 (77% of coaches) or 3 sessions per week (71%), whereas fewer coaches (41% and 35%) had progressed to 3 or 2 sessions per week, and 12–15 repetitions.

**Table 3:** Description of which exercises that were used by players in the adductor group

	Use among players (*n* = 64)				
	Yes	No, have not tried	No, too hard/difficult	No, too easy	No, had pain
Copenhagen adduction, long lever*	37 (57.8)	19 (29.7)	4 (6.3)	0 (0.0)	4 (6.3)
Copenhagen adduction, short lever*	40 (62.5)	16 (25.0)	0 (0.0)	5 (7.8)	3 (4.7)
Side-lying adduction	46 (71.9)	9 (14.1)	1 (1.6)	6 (9.4)	2 (3.1)
Adductor squeeze, straight legs	26 (40.6)	33 (51.6)	1 (1.6)	3 (4.7)	1 (1.6)
Adductor squeeze, bent knees	26 (40.6)	34 (53.1)	0 (0.0)	3 (4.7)	1 (1.6)

Numbers represent *n* players and (percent)

*11 players (17.2%) had not used either of the two Copenhagen adduction exercises

### Use of Programme Material

Printed material, digital material, films, and practical workshops were generally highly valued by coaches in the randomised groups with ratings from 5 to 7 on a 1–7 Likert scale (Table 4). However, 20 (54%) of the coaches did not access the digital material.

**Table 4:** Use of programme material and how coaches accessed the digital material

	Extended *Knee Control* group (*n* = 20)	Adductor group (*n* = 17)
‘How valuable did you find the programme material and instructions?’*		
Printed material, median (IQR), min–max	6.0 (1.0), 3–7	6.0 (1.5), 1–7
Did not access written material, *n* (%)	2 (10.0)	0 (0.0)
Digital material (pdf)	5.0 (2.5), 2–7	6.0 (3.5), 2–7
Did not access digital material (PDF), *n* (%)	7 (35.0)	8 (47.1)
Films on website	5.5 (4.3), 2–7	5.0 (3.0), 3–7
Did not access films on website, *n* (%)	6 (30.0)	8 (47.1)
Practical workshop	6.0 (3.0), 2–7	7.0 (1.0), 4–7
Did not take part in practical workshop, *n* (%)	5 (25.0)	1 (5.9)
Telephone contact with physiotherapist	4.0 (4.5), 1–7	4.0 (2.3), 2–7
Did not have telephone contact, *n* (%)	8 (40.0)	7 (41.2)
*‘If you accessed digital material, what media did you most often use?’*^†^		
Mobile phone	7 (35.0)	3 (17.6)
Tablet	3 (15.0)	0 (0.0)
Computer	5 (25.0)	3 (17.6)
Did not use any digital material (pdf or films)	9 (45.0)	11 (64.7)

*Values are given on a 1–7 Likert scale, where 1 represented “not valuable”, 4 “rather valuable” and 7 “most valuable”. Values are presented as the median, interquartile range, and min–max values or *n* (%) coaches

^†^Values represent number of coaches (%)

### Other Measures to Prevent Injuries

To prevent injuries, 29–41% of coaches had used complementary training, and 37–62% of players had used strength training with weights (Table 5). Players also used protective equipment (52–62%) and taping (20–38%) to a high extent. 59% of coaches in the adductor group reported use of the (original) *Knee Control* programme.

**Table 5:** Use of different interventions to prevent injuries

	Extended *Knee Control* group		Adductor group		Comparison group	
	*n* = 20 coaches	*n* = 120 players	*n* = 17 coaches	*n* = 63 players	*n* = 24 coaches	*n* = 105 players
Strength training with weights	1 (5.0)	59 (49.2)	5 (29.4)	39 (61.9)	3 (12.5)	39 (37.1)
Complementary training (fitness training, yoga, group training)	8 (40.0)	32 (26.7)	7 (41.2)	19 (30.2)	7 (29.2)	37 (35.2)
Balance training	4 (20.0)	23 (19.2)	5 (29.4)	15 (23.8)	5 (20.8)	15 (14.3)
Taping	6 (30.0)	24 (20.0)	11 (64.7)	17 (27.0)	16 (66.7)	40 (38.1)
Protective equipment (such as shin guards)	4 (20.0)	70 (58.3)	10 (58.8)	33 (52.4)	16 (66.7)	65 (61.9)
Advice from physiotherapist, clinician, naprapath	8 (40.0)	20 (16.7)	2 (11.8)	17 (27.0)	7 (29.2)	29 (27.6)
Education (in nutrition, sleep, or training)	0 (0.0)	8 (6.7)	1 (5.9)	3 (4.8)	5 (20.8)	22 (21.0)
Knee Control programme	N/A	N/A	10 (58.8)	23 (36.5)	21 (87.5)	82 (78.1)
Hamstring strengthening	N/A	N/A	14 (82.4)	29 (46.0)	15 (62.5)	52 (49.5)
Hip/groin strengthening	3 (15.0)	15 (12.5)	N/A	N/A	10 (41.7)	32 (30.5)
One-legged knee squats	N/A	N/A	9 (52.9)	N/A	15 (62.5)	N/A
Two-legged knee squats	N/A	N/A	12 (70.6)	N/A	19 (79.2)	N/A
Core strengthening	N/A	N/A	13 (76.5)	N/A	19 (79.2)	N/A
Lunges	N/A	N/A	13 (76.5)	N/A	19 (79.2)	N/A
Jump/landing technique	N/A	N/A	11 (64.7)	N/A	13 (54.2)	N/A
Plyometrics	N/A	N/A	10 (58.8)	N/A	10 (41.7)	N/A
Strength training with elastic tubes	N/A	N/A	4 (23.5)	N/A	7 (29.2)	N/A
Other IPEP	0 (0.0)	8 (6.7)	2 (11.8)	2 (3.2)	3 (12.5)	17 (16.2)

Data denotes *n* (%) who used the respective interventions. N/A (not applicable) represent questions where this specific group of coaches or players did not receive the question

IPEP, Injury Prevention Exercise Programme; N/A, not applicable

### Adverse Events

In the three groups, 11% (extended *Knee Control* group), 20% (comparison group) and 23% (adductor group) of players had experienced pain during the injury prevention exercises and used different coping strategies to manage pain (Additional file 3: Table S1). Among players who experienced pain, the median pain intensity according to a numerical rating scale (0–10) was rated as 3.0 (comparison group), 3.5 (adductor group), and 5.0 (extended *Knee Control* group). For players with pain, coaches predominantly used alternative exercises, or changed to easier exercises or fewer repetitions. No other adverse events were reported to the researchers during or after the study.

### Perceptions of the Respective Programmes

Players liked that the programmes reduced their risk of injury and that their performance of the exercises improved over time. Players in the extended *Knee Control* group and the adductor group also appreciated that the exercises could be varied over time (Table 6). Players disliked that they had less time for football training and that the exercises were boring.

**Table 6:** Description of what the players liked/did not like with their respective programme

	Extended *Knee Control* group	Adductor group	Comparison group
	(*n* = 118)	(*n* = 60)	(*n* = 104)
*Liked*			
The exercises reduced my risk of injury	107 (90.7)	48 (80.0)	95 (91.3)
I got better at performing the preventive exercises	56 (47.5)	34 (56.7)	39 (37.5)
The exercises could be varied over time	53 (44.9)	25 (41.7)	25 (24.0)
Some exercises could be done with a partner	40 (33.9)	15 (25.0)	39 (37.5)
We used the ball during some exercises	36 (30.5)	N/A	23 (22.1)
We used equipment during some exercises	36 (30.5)	N/A	13 (12.5)
Structured warm-up	28 (23.7)	10 (16.7)	48 (46.2)
Exercises were a break from normal training	24 (20.3)	17 (28.3)	19 (18.3)
I became a better player from doing preventive exercises	9 (7.6)	4 (6.7)	15 (14.4)
We could compete in some exercises	6 (5.1)	N/A	8 (7.7)
Nothing, I did not like the programme/exercises	7 (5.9)	4 (6.7)	3 (2.9)
*Disliked*			
We had less time for football training	58 (49.2)	12 (20.0)	48 (46.2)
The exercises were boring	45 (38.1)	15 (25.0)	49 (47.1)
The programme was too long	21 (17.8)	1 (1.7)	3 (2.9)
The exercises did not have anything to do with football	6 (5.1)	3 (5.0)	10 (9.6)
The exercises were too easy	5 (4.2)	1 (1.7)	10 (9.6)
I got pain during preventive training	5 (4.2)	6 (10.0)	6 (5.8)
I did not understand why I should do preventive training	1 (0.8)	4 (6.7)	3 (2.9)
The exercises were too hard/difficult	0 (0.0)	1 (1.7)	0 (0.0)
The programme was too short	0 (0.0)	4 (6.7)	7 (6.7)
Nothing, I liked the programme/exercises	38 (32.2)	28 (46.7)	32 (30.8)

Numbers are *n* (percent). N/A (not applicable) represent questions where this specific group of players did not receive the alternative. Each player could give multiple answers

## Discussion

Overall adherence with the IPEPs was high, but utilisation fidelity was lower regarding the number of repetitions per exercise and progression, the latter only regarding the adductor (progression of number of repetitions) and comparison group (progression through other exercises). The results are positive, considering that the study was carried out during the first year of COVID-19. Players liked that the IPEP reduced injuries and that they got better at performing the exercises but disliked that they had less time for football and that the exercises were boring. Not all coaches accessed the digital material, suggesting that programme material needs to be disseminated in different forms.

### Adherence with the Programmes

Cumulative utilisation, utilisation frequency, and duration fidelity indicated high adherence to the programme protocols in the extended *Knee Control* group and the comparison group (where teams to a large extent used the original *Knee Control* programme). Recommendations for the adductor programme differed from the other groups, with higher recommended frequency during pre-season than during the competitive season, and progressive increases in number of repetitions. Accordingly, cumulative utilisation, utilisation frequency, and duration fidelity differed from the other groups but also indicated quite high adherence in the adductor group. Despite lower recommended utilisation frequency compared to the other groups, the adductor group reported lower utilisation frequency than expected, with one training session per week on average, which was similar to the frequency recommended during the competitive season but less than the 2 or 3 times/week recommended during pre-season. Utilisation fidelity is questionable in all three groups. The majority of teams and players did 1–10 repetitions per exercise, which probably is fewer than required for most exercises, except for the heavy eccentric *Nordic Hamstring Exercise* in the extended *Knee Control* group, and *Copenhagen Adduction* in the adductor strength programme. The number of repetitions were also lower than recommended in the programme recommendations for *Knee Control* (where 8–15 repetitions are recommended and has shown positive effect on injury rates) [1, 23] and for extended *Knee Control* (where 30–60 s, exceeding 10 repetitions for most exercises also with the shortest duration) are recommended [19]. Up to 12 repetitions is often recommended for strength training aiming for muscle hypertrophy, and at least 10 for muscular endurance [24]. Considering the neuromuscular focus in most exercises, where neuromuscular adaptations with improved muscle action are aimed for, more than 10 repetitions is probably needed to elicit maximum training stimuli. However, for a full picture of the training stimuli and potential for effect, we would also have needed information about the number of sets per exercise [25] as well as the amount of rest between exercises or sets, time under tension, range-of-motion during exercises and whether training was until failure or not [26]. It was positive that 90% of coaches and 78% of players in the extended *Knee Control* group had used different exercises for variation and more advanced exercises over time (30% of coaches), whereas progression and variation in the comparison group was less frequent. Teams in the adductor group seem to have adhered poorly to the prescribed load. The teams were supposed to start at the highest level and only use easier variants when necessary. However, 72% of players reported use of the easiest dynamic variant, side-lying adduction, and 17% had used neither of the Copenhagen adduction long or short lever exercises. Additionally, few coaches in the adductor group reported that they had progressed the number of repetitions according to recommendations. One explanation could be that the season planning was altered by the COVID-19 pandemic. It could also be that the exercises and the programme progression plan was too ambitious and difficult to adhere to for amateur players, since the programme has previously only been evaluated among sub-elite male players [3]. These results, with modified training dosage and progression, are in line with the results of a study on the *Adductor Strengthening Programme* among professional football players in Norway [27].

In the main trial we showed differences in programme preventive effects between groups. Lower incidences of injuries to the hamstring, knee, and ankle combined were seen in the extended *Knee Control* group versus the comparison group, and a lower injury prevalence compared to both groups [19]. These differences are interesting considering that 78–88% of coaches and players in the comparison group and 37–59% in the adductor group indicated that they had used the (original) *Knee Control* programme during the study. These groups may not have used the *Knee Control* programme according to recommendations; or alternatively, the extended *Knee Control* programme may have induced higher training effects, owing to the more intense exercise variations available. The adductor group was small, however, rendering low power to detect differences between groups. We know from earlier studies that coaches modify the content of the *Knee Control* programme [13, 14], and we specifically asked about modifications in the present study. Only one fourth of responding coaches (*n* = 5) in the extended *Knee Control* group had modified exercises (added or replaced) and two of those had done so due to pain experienced by individual players. It is also interesting that some teams in all three groups had embedded the IPEP into football training, or that the extended *Knee Control* group and the comparison group used it after training, possibly to improve player adherence [28]. In summary, all three interventions faced challenges with adherence, despite their different content, set-up, extent and training recommendations. This suggests that other factors than the programme content or programme material per se are important to address to enhance adherence. For instance, to focus on the end-users (coaches and athletes) and their need for support and understanding about how to attain the intended training effects. Future strategies could be aimed at improving support and education to coaches, players and parents to enhance motivation for injury prevention as well as adherence to IPEPs.

### Perceptions of the Programmes and Use of Programme Material

Considering our digitalised society, it was surprising that half of all coaches in the randomised groups did not access the provided digital material. This emphasises that one-size-fits-all solutions for programme dissemination are less likely to work. We also need to consider the non-negligible part of players in extended *Knee Control* and the comparison group who perceived the programmes as being too long and boring. Fewer players in the adductor group reported these barriers, suggesting that a shorter and less frequently used programme may be more accepted among players. However, before a multi-component programme is abbreviated or recommended with a lower utilisation frequency, the potential impact on preventive efficacy needs consideration as current evidence indicates that high compliance positively associates with greater preventive effects [29]. Player motivators for IPEP merit further investigation to learn more about how to support and motivate players.

Some players (11–23% per group) experienced pain during the exercises, which should be given further attention in future studies, especially since the median pain scores were rather high (5 on a 0–10 numerical rating scale for the players in the extended *Knee Control* group with pain). We did not collect data on reasons for pain, and this could include anything from aggravation of ongoing injury-related pain to delayed onset muscle soreness. In an earlier study [23] we found that female players who used the *Knee Control* programme reported gradual onset knee pain to higher extent than the control group. Since many players are pubertal or post-pubertal, pain may be related to rapid growth and aggravated, but not caused, by IPEP training. Additionally, too rapid progression of heavy eccentric exercises such as the *Nordic Hamstring Exercise* and the *Copenhagen Adduction* may induce delayed onset muscle soreness or provoke pain in players with ongoing posterior thigh or hip/groin complaints.

### Final Developed Version of Knee Control+

The extended *Knee Control* programme was feasible for use across one season. Since the programme’s use was evaluated within a randomised trial, similar adherence when used in the real-world context or other settings cannot be taken for granted. Since the extended *Knee Control* programme does not include exercises specifically targeting the hip/groin we have now merged the adductor programme into the extended *Knee Control* programme, considering that the exercises in the adductor programme were found feasible. Additionally, a significant number of teams in the adductor group had spontaneously adopted the (original) *Knee Control* programme, which suggests that merging programmes together is feasible from the coaches’ point of view as well. We call this new version (with adductor exercises added and only one exercise with knee squats) *Knee Control*+ (https://liu.se/forskning/swipe/knakontroll-plus) and plan further studies on the long-term use of the programme. This is in line with the inclusion of the *Adductor Strengthening Programme* into the *11*+ programme [30].

### Methodological Considerations

A strength of this study is the comprehensive approach to studying different aspects of adherence from both the perspective of coaches/teams and players. Another strength was the detailed description of how IPEPs were used, rather than simply reporting as used or not used.

Some limitations also need to be mentioned. First, the potential influence of the pandemic, with modification of the adductor programme and avoidance of close contact, as well as the extended pre-season and shortened competitive season, should be considered. We also had larger than expected drop-out from coaches who initially agreed to participate with their teams, which may have affected the representativity of participants. Additionally, adherence rates may have been affected by recommendations to stay home when feeling symptoms potentially related to a COVID-19 infection. The response rate was rather low but in line with studies in youth players [23, 31, 32] and similar between the three groups, and we believe it is unlikely that the survey response rate affected the adherence outcomes differently in the three groups.

Second, data were self-reported, and players and coaches may have tried to present as positive responses as possible. Responding via questionnaires may also limit the possibility to describe how the programmes were used if they were not used in a similar way every time. Due to the pandemic and limited study resources we were not able to conduct site visits to teams and observe programme and exercise fidelity, which could have supplemented the self-reports. We also lack detailed information about the experiences of pain during IPEP training, and pain location and intensity, which warrants further study. Additionally, we could not ask players in the comparison group specific questions about how they used their IPEP, since not all players used the same IPEP.

Third, the questionnaires have not been formally validated. However, we have used similar questions in previous studies without players or coaches raising concerns about their relevance. Additionally, some questions were highly influenced by another study in football [33], but adapted to the Swedish context and for use at different timepoints. Hence, we believe that the questions have high face validity.

Fourth, the question about use of protective equipment and taping showed unexpectedly few users of protective equipment whereas more players than expected used taping. Since these were closed-ended questions, we have no additional information on this matter, which is a limitation of the study.

Fifth, due to low sample size we were not able to analyse and compare adherence in sub-groups of players.

## Conclusions

We conclude that adherence was generally high regarding cumulative utilisation, utilisation frequency, and duration fidelity. Players liked that the programmes reduced their injury risk and that their performance of the exercises improved during the season. Almost one-fifth of all players reported experiencing some pain during preventive exercises and this merits further attention. Half of all coaches did not use the provided digital programme material indicating that different means of programme material dissemination are needed to accommodate different coaches’ preferences. Coaches also used complementary training and players used strength training with weights to prevent injuries. This sub-study was part of a randomised trial, and it cannot be taken for granted that adherence would be similar in a real-world context.

## Supplementary Information

**Additional file 1**. Extended *Knee Control* programme folder.

**Additional file 2**. Adductor strength programme, original version and Adductor strength programme, alternative exercises that were added due to the pandemic

**Additional file 3: Table S1**. Description of how coaches and players dealt with pain during training.

## Data Availability

All data generated or analysed during this study are included in this published article [and its additional files].

## Abbreviations

COVID-19: Coronavirus Disease 2019
IPEP: Injury prevention exercise programme
SD: Standard deviation
