# Supplementary material for: Adherence to Injury Prevention Exercise Programmes in Amateur Adolescent and Adult Football: A Detailed Description of Programme Use from a Randomised Study
Source: Sports Med Open. 2023 Jul 15;9:57. doi: 10.1186/s40798-023-00608-1 (PMC10349794; doi:10.1186/s40798-023-00608-1)
Supplement: Supplementary file 1 — Additional file 1. Extended Knee Control programme folder. [file 40798_2023_608_MOESM1_ESM.pdf]

Sports Medicine – Open. Adherence to injury prevention exercise programmes in amateur adolescent and adult football— A detailed description of programme use from a randomised study. Lindblom H, Waldén M, Hägglund M.

Affiliation for corresponding author: Unit of Physiotherapy, Department of Health, Medicine and Caring Sciences, Linköping University, Linköping, Sweden. Sport Without Injury ProgrammE (SWIPE), Department of Health, Medicine and Caring Sciences, Linköping University, Linköping, Sweden
E-mail address for corresponding author: hanna.lindblom@liu.se

Hanna Lindblom, Markus Waldén and Martin Hägglund declare that they have no competing interests.

The study was funded by grants from the Swedish Research Council Ref. no. 2018-03135 and Region Östergötland Ref. No. 922771.

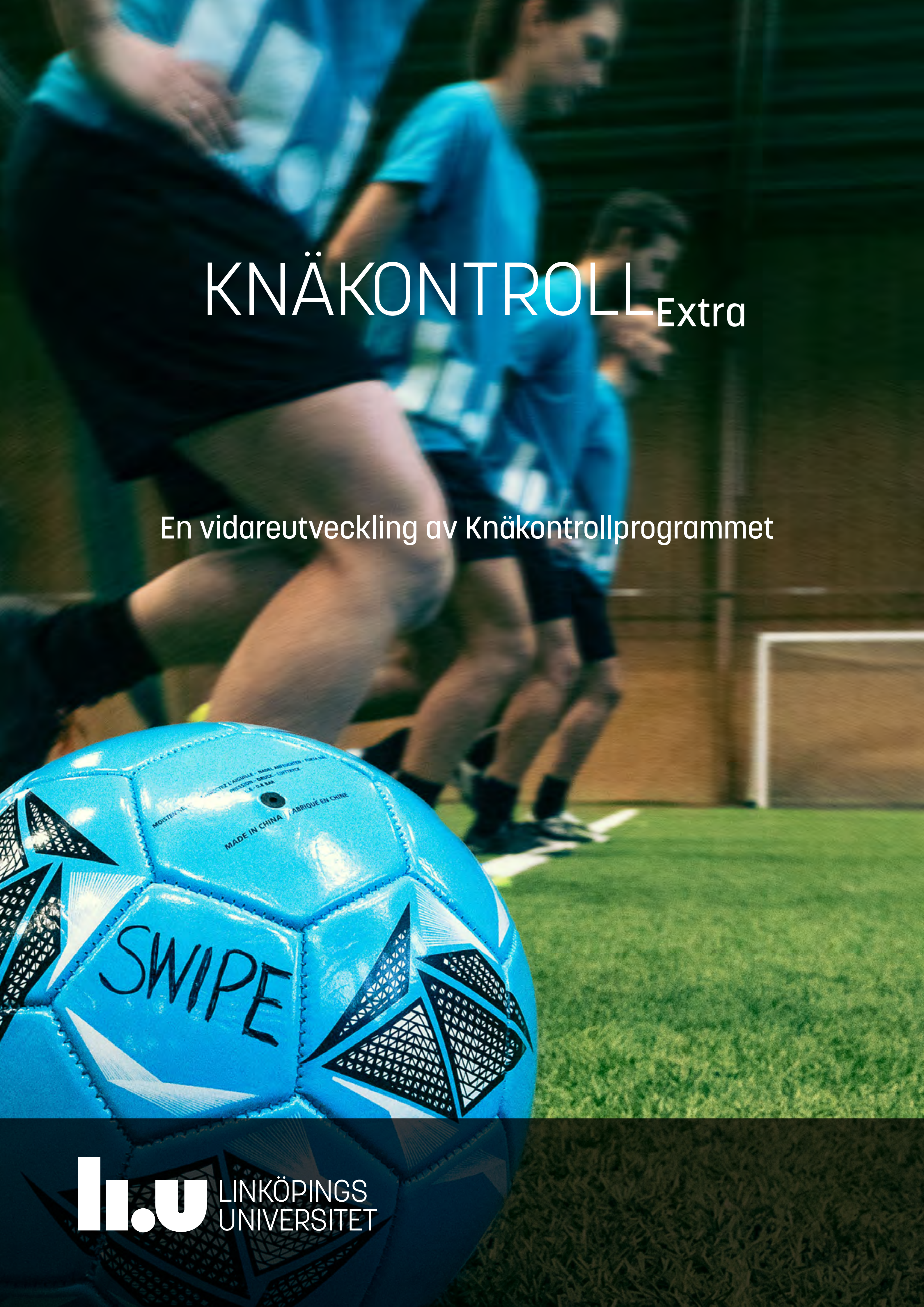

KNÄKONTROLL Extra

En vidareutveckling av Knäkontrollprogrammet

LINKÖPINGS UNIVERSITET

Innehåll

Träningsprogrammet Knäkontroll 4
Vidareutveckling av Knäkontroll 4
Arbetsgruppen som varit delaktig vid framtagandet av Knäkontroll$_{Extra}$ 4
Övergripande upplägg 5
- När du väljer övningar 5
- Korrekt och felaktigt utförande av övning 6
- Exempel på träningsupplägg 7

Generell uppvärmning 8
Övningsbank 12
- 1. Enbensknäböj 12
- 2. Baksida lår (hamstrings) 15
- 3. Knäböj på två ben 18
- 4. Bålstyrka 21
- 5. Utfall 24
- 6. Hopp/landning 27

Träningsprogrammet Knäkontroll

Knäkontrollprogrammet utvecklades ursprungligen 2005 och syftar till att förebygga skador och förbättra prestationsförmågan. Hos flickfotbollsspelare som använde programmet minskade risken för allvarlig knäskada (främre korsbandsskada) med 64% i en vetenskaplig studie genomförd i samarbete mellan Linköpings universitet, Svenska Fotbollförbundet och försäkringsbolaget Folksam med Martin Hägglund och Markus Waldén som ansvariga forskare (Waldén et al., British Medical Journal 2012;344:e3042,).

Rekommendationen är att starta med övningarna från 10-12 år och fortsätta träna programmet regelbundet i samband med uppvärmningen inför fotbollsträning genom hela fotbollskarriären.

Vidareutveckling av Knäkontroll

Allteftersom Knäkontroll har blivit mer spritt och använt inom fotboll har också många tränare börjat uttrycka önskemål om mer variation i övningarna. Tränare för de yngsta spelarna beskriver spelare som ibland har svårt att klara av en del av övningarna, medan tränare för äldre ungdomslag och seniorlag snarare önskar ett, mer utmanande program.

Knäkontroll$_{\text{Extra}}$ utvecklades för att tillgodose dessa önskemål: programmet innehåller både lättare och mer utmanande övningar och ger ökade möjligheter för tränaren att anpassa övningar till sitt eget lag. Programmet är framtaget och pilottestat inom SWIPE, Sport Without Injury ProgrammE, vid Linköpings universitet.

Arbetsgruppen som varit delaktig vid framtagandet av Knäkontroll$_{\text{Extra}}$

Martin Hägglund, leg sjukgymnast, professor, Linköpings universitet, **Hanna Lindblom**, leg sjukgymnast, medicine doktor, Linköpings universitet, **Markus Waldén,** överläkare, medicine doktor, Linköpings universitet, Svenska Fotbollförbundets medicinska kommitté, **Sofi Sonesson,** leg sjukgymnast, medicine doktor, Linköpings universitet, **Anne Fältström**, leg sjukgymnast, medicine doktor, Linköpings universitet, Svenska Fotbollförbundets medicinska kommitté, **Tania Nilsson**, leg sjukgymnast, förbundssjukgymnast ungdomslandslag Sverige, Djurgårdens IF, **Mariann Gajhede Knudsen**, leg sjukgymnast, förbundssjukgymnast Norge, fotbollstränare (UEFA A-licens), **Annica Näsmark**, leg sjukgymnast, förbundssjukgymnast damlandslaget i fotboll, Svenska Fotbollförbundets medicinska kommitté, **Mia Ryding-Ederö**, leg sjukgymnast, Linköping, Linköpings Fotboll Club.

Övergripande upplägg

Uppvärmning genomförs inför varje träning och match: ca 5 minuter, gärna med löpövningar under förflyttning. Dessa kan varieras med och utan boll.

Knäkontroll$_{Extra}$övningar genomförs lämpligen inför varje träning som en del i uppvärmningen under **10-15 minuter**, men kan även vävas in i träningen eller göras efter träning om detta passar lagets träningsupplägg bättre. Programmet består av **6 olika grundövningar** som vardera görs under **30-60 sekunder** (motsvarar ca 8-15 repetitioner per övning) i **2 set**. Spelarna ska bli trötta av övningarna men klara att göra alla repetitioner med god teknik.

Varje grundövning finns i 10 olika varianter. Av praktiska skäl brukar oftast hela laget göra samma variant av övningen, men om möjligt får gärna en individuell anpassning ske. Fortsätt gärna med **samma övningsvarianter** tills spelarna lärt sig dessa innan ni byter variant.

Tänk på grundtekniken i samtliga övningar: knä och fot i samma riktning, god bålstabilitet, höftbredd mellan fötterna och mjuka kontrollerade landningar. I programmet finns även blå rutor med tips på hur du som tränare enkelt kan instruera spelarna i respektive övning.

När du väljer övningar

- Välj övningar som är **lagom svåra** – spelarna ska kunna göra övningarna med god teknik. Övningarna är beskrivna från lätta till mer utmanande övningar. Parövningar och gummibandsövningar är inte nödvändigtvis svårare än de individuella övningarna men kan läggas in som variation.
- **Ta med en variant av samtliga sex grundövningar** (enbensknäböj, baksida lår, knäböj på två ben, bålstyrka, utfallssteg och hopp/landning).
- Använd gärna **gummibandsövningar** som variation för att arbeta mer med muskulatur på höftens utsida och rumpa.
- Är det dåligt väder kan stående övningar vara att föredra. Ett tips är annars att **använda sittunderlag** för att undvika att bli kalla och blöta i samband med liggande övningar.

Knäkontroll Extra

Korrekt och felaktigt utförande av övning

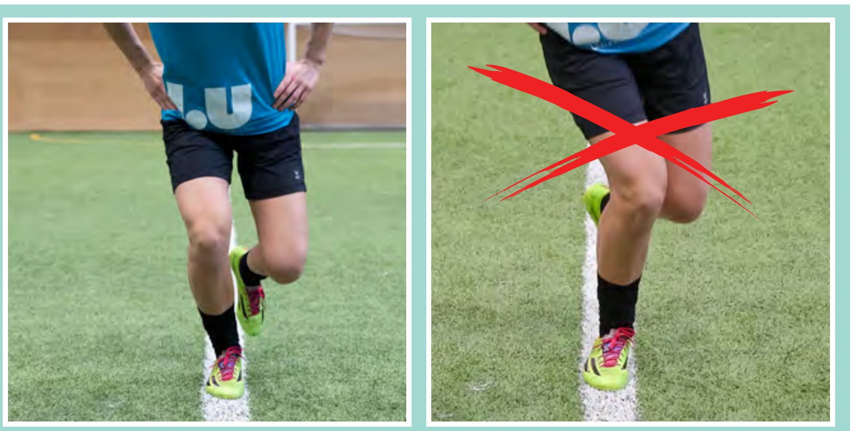

Enbensknäböj – fot och knä ska peka i samma riktning samt att höften ska vara stabil.

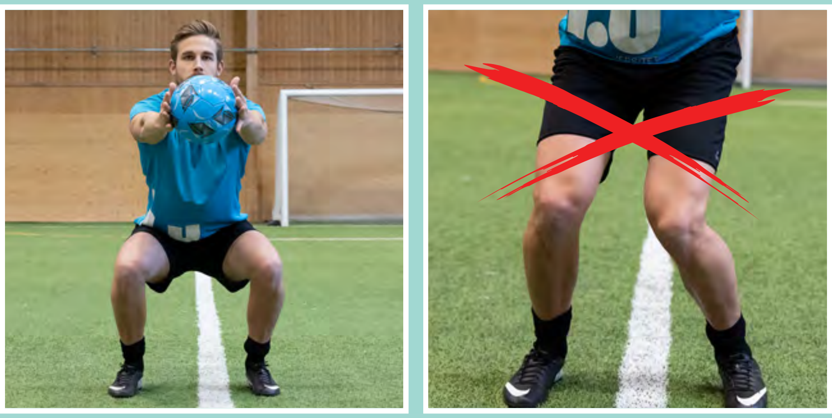

Knäböj på två ben – hela fötterna ska vara i marken och knäna ska peka rakt fram, inte inåt.

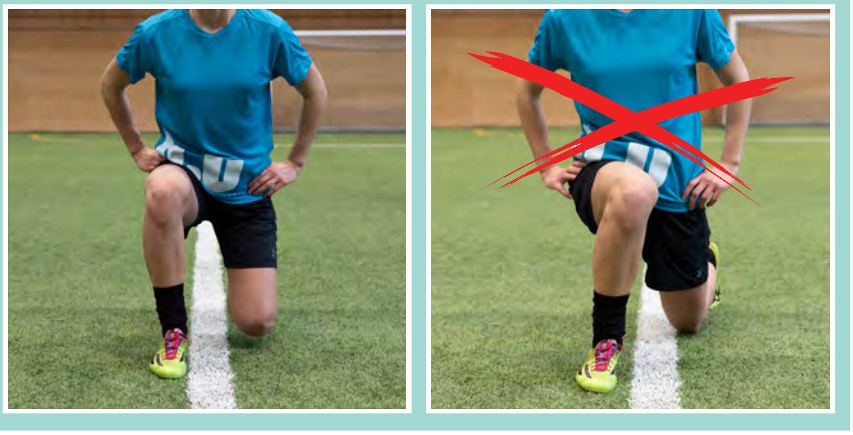

Utfall – fötterna står som på räls och kroppen rör sig rakt upp och rakt ner, överkroppen hålls upprätt och utan att luta åt sidan.

Exempel på träningsupplägg

Löpuppvärmning 5 minuter.
Löpning framåt, jojo-jogg, slalom, indian-hopp, sidledshopp med låg tyngdpunkt.

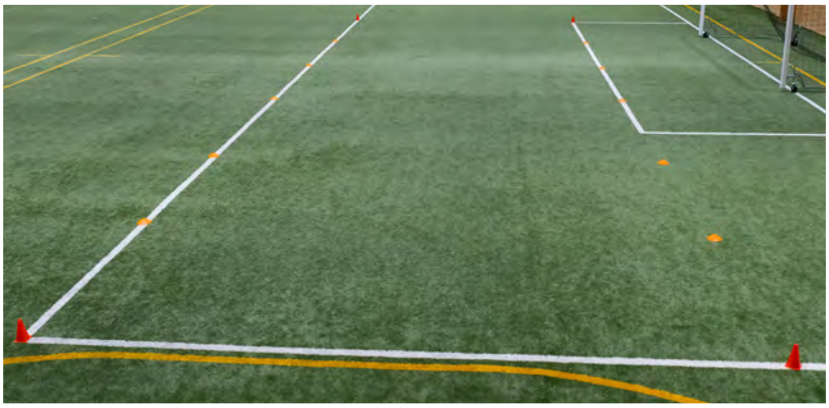

Enbensknäböj 1J
30-60 sekunder x 2.

Baksida lår (hamstrings) 2F
30-60 sekunder x 2.

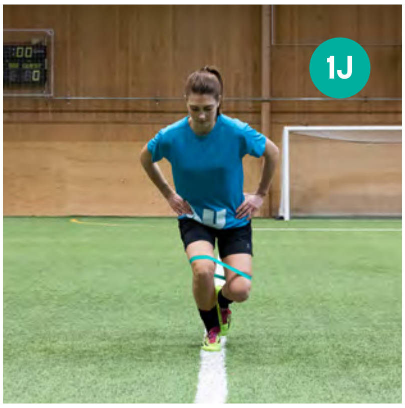

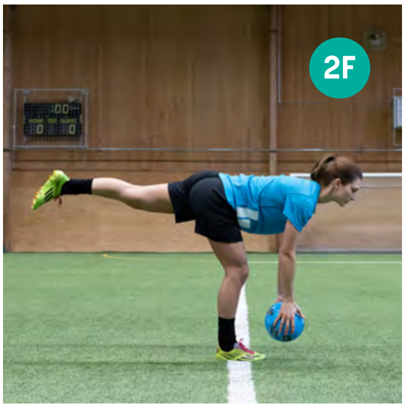

Knäböj på två ben 3C
30-60 sekunder x 2.

Bålstyrka 4I
30-60 sekunder x 2.

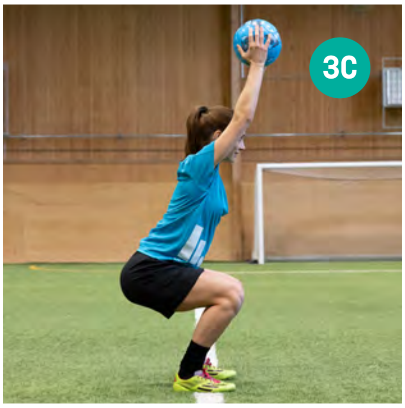

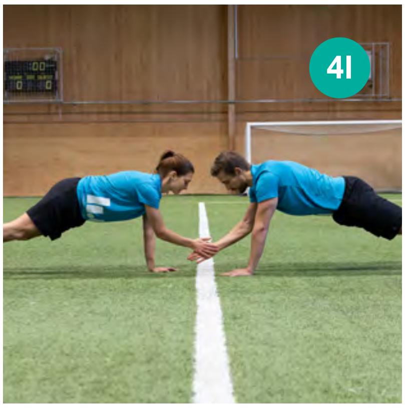

Utfall 5G
30-60 sekunder x 2.

Hopp/landning 6G
30-60 sekunder x 2.

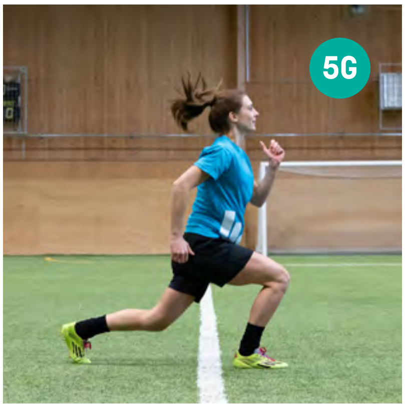

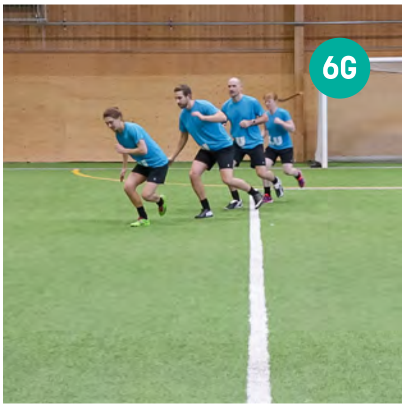

Generell uppvärmning

Genomför en löpuppvärmning i ca 5 minuter (1 minut per övning) innan Knäkontroll+övningar. Nedan ges ett förslag på övningar att välja bland, men det är även möjligt att välja andra övningar. Välj ca fem övningar.

Uppvärmningen kan göras på en fotbollsplan eller på sidan av planen om denna är upptagen. För löpuppvärmningen är det bra med en sträcka om ca 20 m att springa och med en bredd om ca 10 m för att även få plats för sidoförflyttningar. Det måste även finnas lite utrymme på utsidan av konerna när spelarna springer tillbaka till startpositionen. Spelarna arbetar två och två över planen. **Det går bra att även använda bollen i övningarna.**

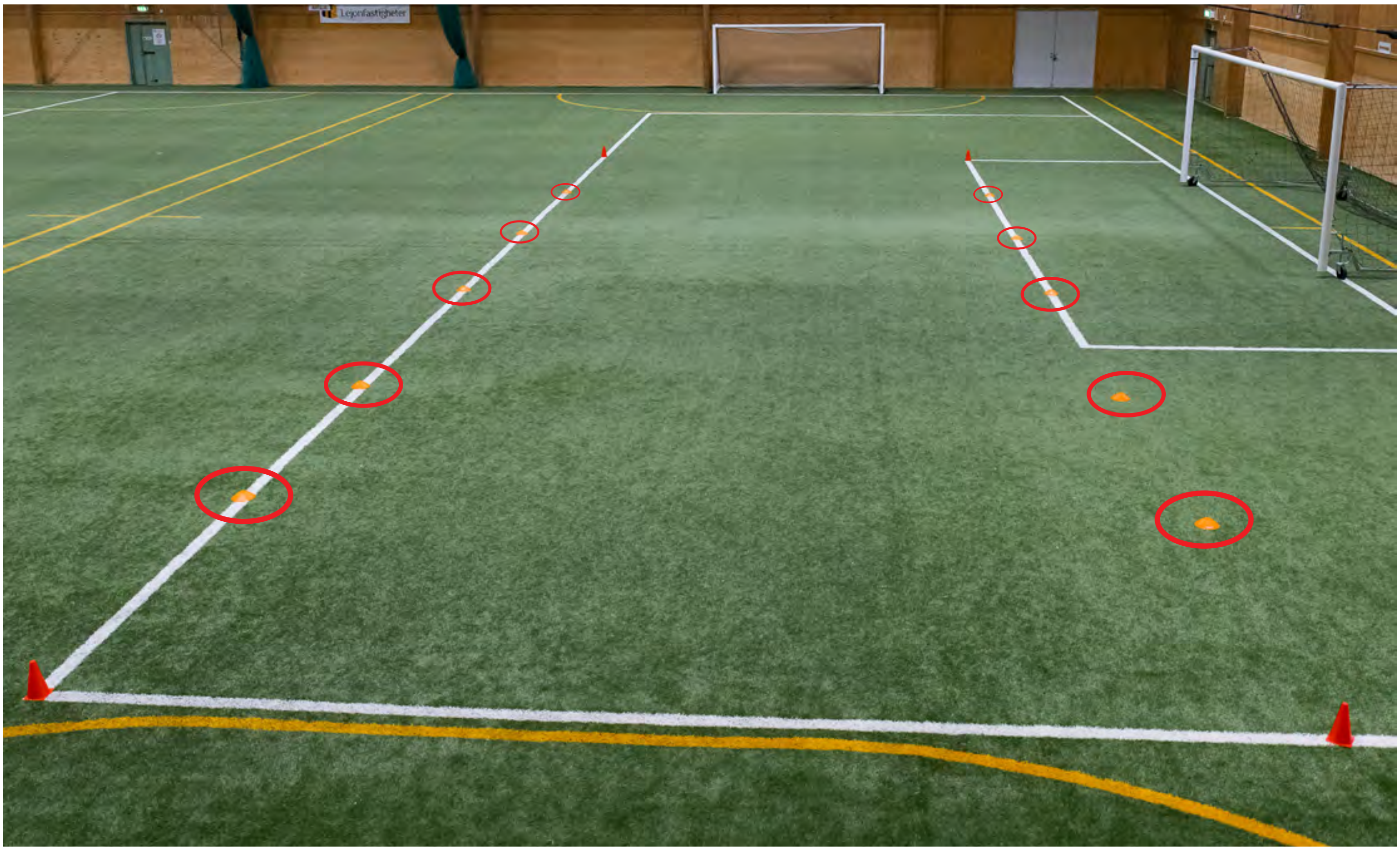

Uppvärmning Knäkontroll Extra

1. Löpning rakt fram, anpassa tempot så att spelarna får upp pulsen.

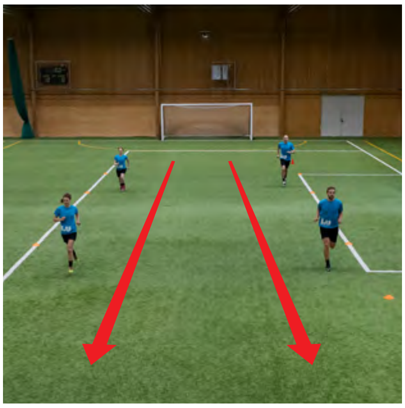
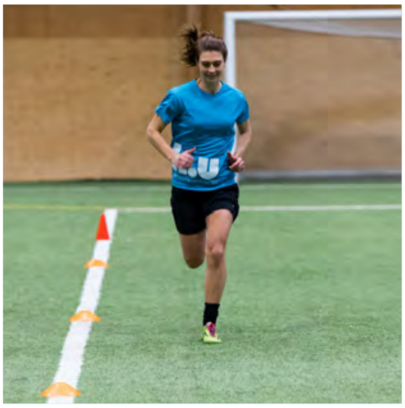

2. Sidledshopp med låg tyngdpunkt, jobba på tå, löpning framåt en konlängd, sidledshopp med låg tyngdpunkt.

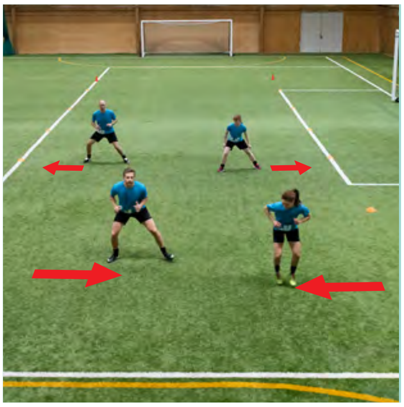
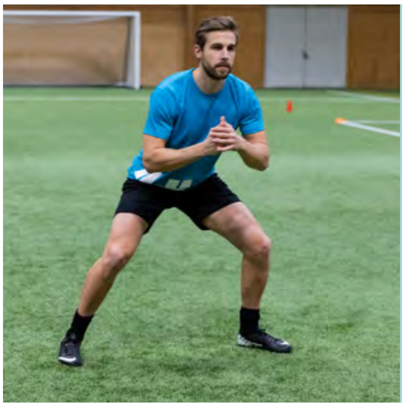

3. Löpning framåt med markerad riktningsändring, som att finta dig förbi en motståndare vid varje kon. Frys rörelsen en kort stund med god kontroll i samband med vändningen.

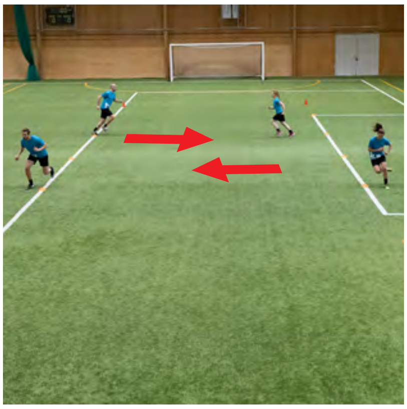
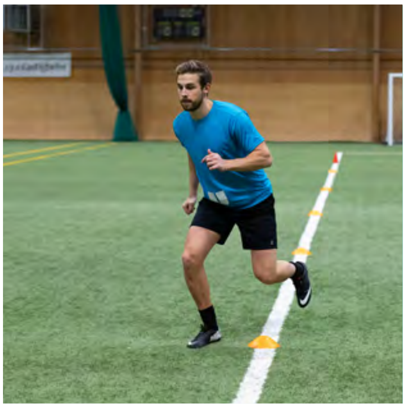

4. Sidledshopp mot mitten och upphopp, där spelarna hoppar upp axel mot axel som i en nickduell och landar med god kontroll, frys landningen en kort stund, löp fram mot nästa kon.

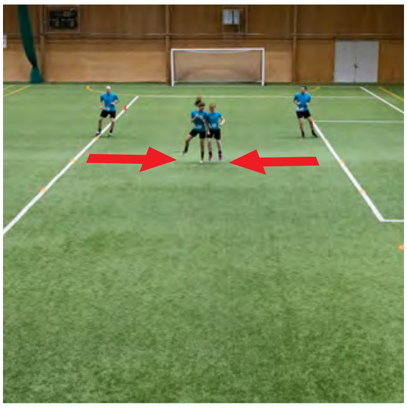
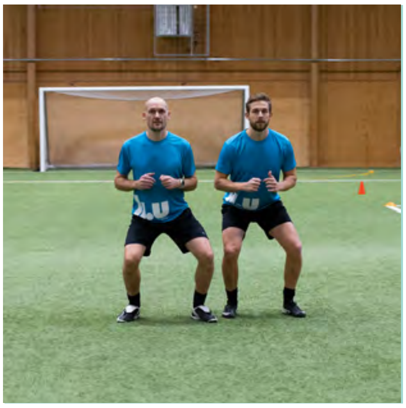

Uppvärmning Knäkontroll Extra

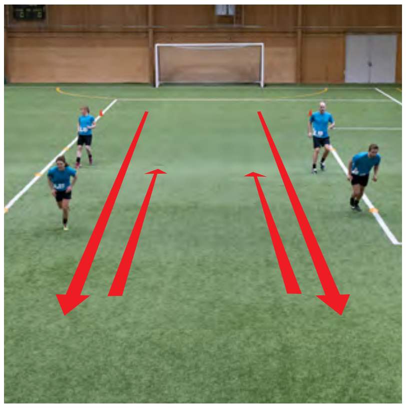
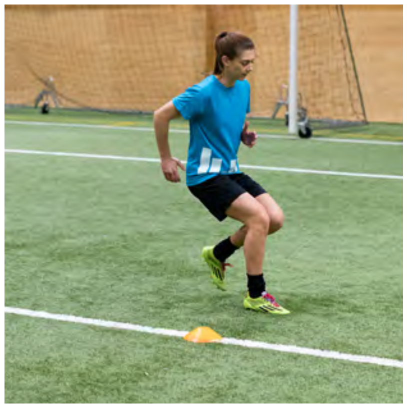

5. ”Jojo-jogg”, jogga två konlängder framåt med korta snabba steg, backa en konlängd, låg tyngdpunkt i vändningarna.

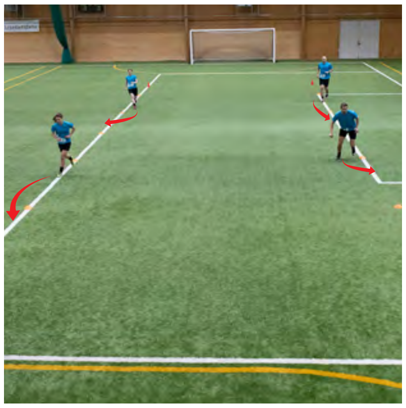
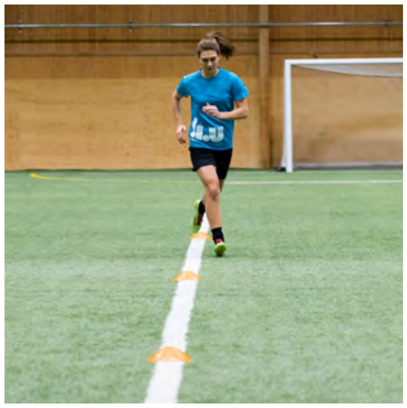

6. Löpning i slalom mellan koner, markera svängarna med en tydlig fotisättning.

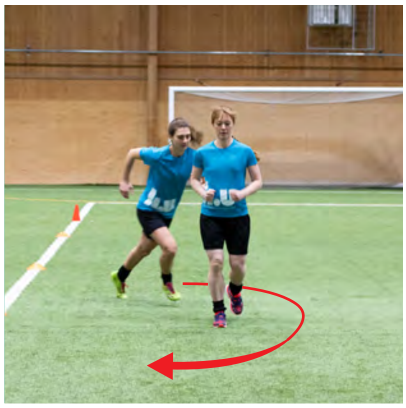
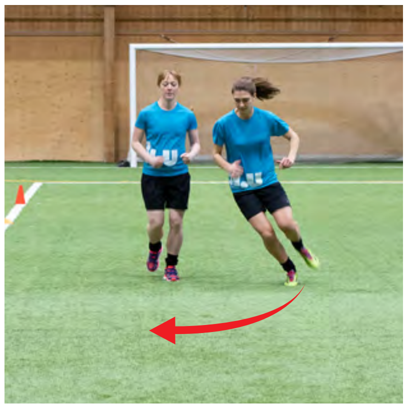

7. Springa runt varandra, den ena spelaren springer rakt framåt samtidigt som medspelaren rundar denne. Spelarna byts av mellan att springa rakt fram och att runda den andre spelaren.

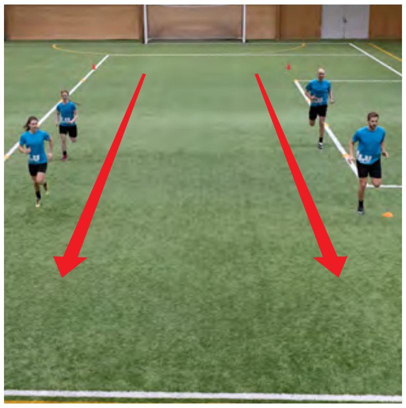
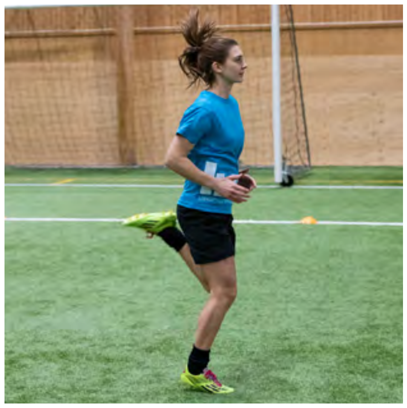

8. Kick i rumpan, spring framåt och försök samtidigt sparka dig själv i rumpan.

9. Indianhopp, hoppa uppåt och framåt över planen med spänst.

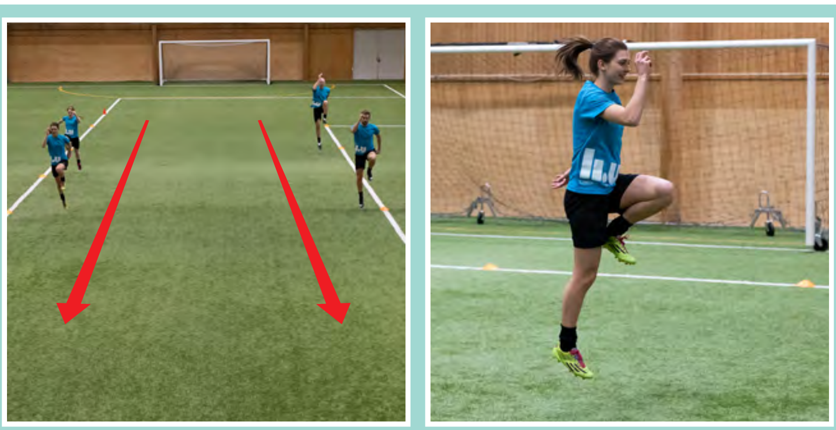

10. Spegling, snabba fötter med förflyttning framåt/bakåt eller i sidled: ena spelaren startar och den andra följer. Spelaren som följer försöker att reagera så snabbt som möjligt och härma den rörelse som den andra spelaren gör.

Snabba fötter, upp på tå

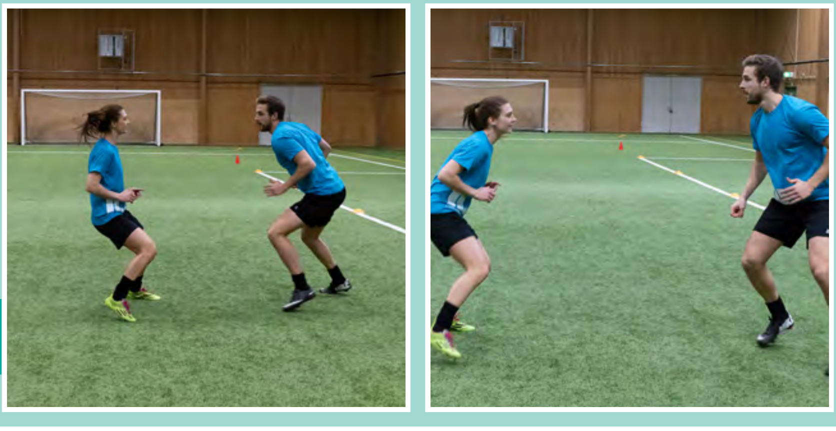

Enbensknäböj

1. Enbensknäböj

Syftet med övningen är att träna balans, benstyrka och att styra knäet rakt över foten. Fokusera på att stående på ett ben göra en långsam rörelse (2 sekunder ner, 2 sekunder upp) med god kontroll över knäet. Tänk att du ska sätta dig ned på en stol. Rörelsen görs med hela ståbenets fot i marken. Det fria benet hålls framför eller bakom ståbenet. Undvik att tippa höften åt sidan.

Instruktionstips

Sätt dig på en stol

Övningar

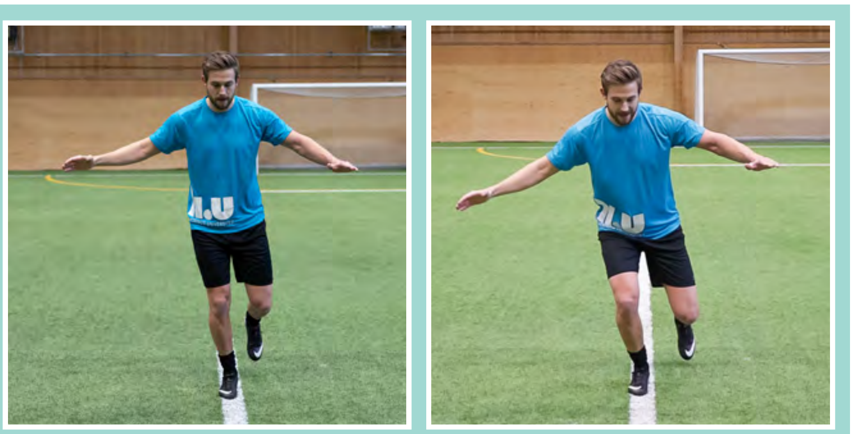

1A Enbensknäböj med armar ut åt sidorna.
Gör ett enbensknäböj och håll armarna ut åt sidorna för att få hjälp med balansen.

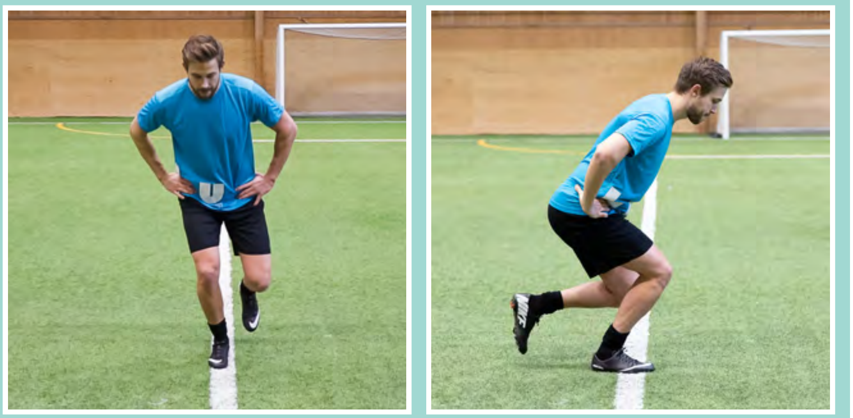

1B Enbensknäböj med händerna vid höfterna.
Gör enbensknäböj i lugnt tempo.

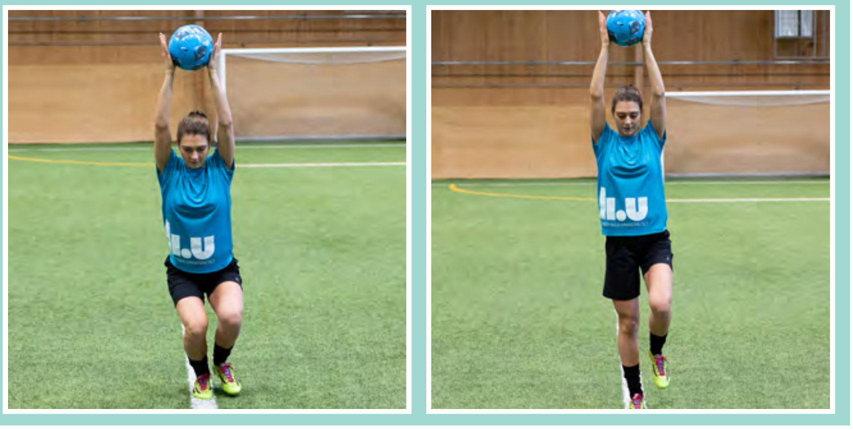

1C Enbensknäböj med raka armar över huvudet.
Håll bollen högt över huvudet.

Enbensknäböj

Övningar

1D Enbensknäböj med fotmarkering.
Gör ett enbensknäböj som för att sätta dig på en stol och för det fria benet mot klockan 12-2-4-6 (höger) eller 12-10-8-6 (vänster).

Instruktionstips

Dutta tårna i gräset

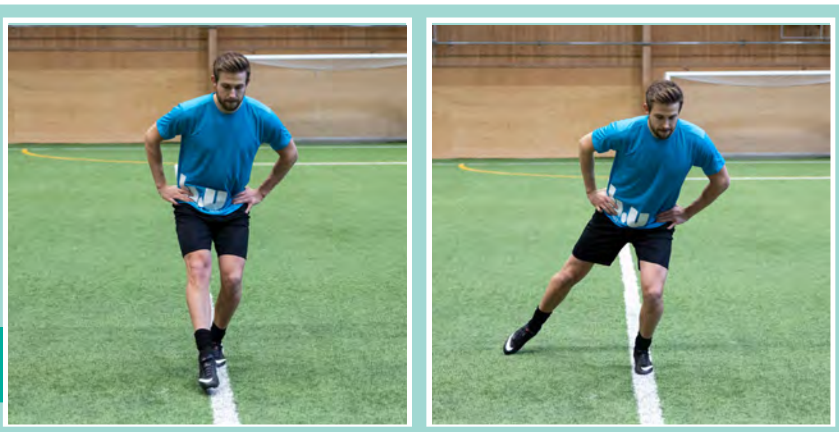

1E Enbensknäböj med diagonalrörelse.
Gör ett enbensknäböj, dutta med bollen på utsidan av foten och för sedan bollen i en diagonal rörelse mot motsatt axel. Följ bollen med blicken.

Instruktionstips

Dutta bollen i marken / för över ena axeln

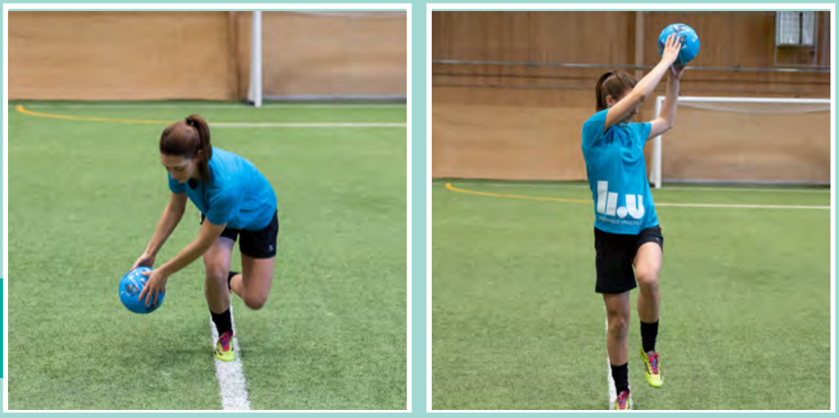

1F Djupt enbensknäböj.
Stegra enbensknäböjet genom att försöka böja ännu djupare med bibehållen kontroll över höft, knä och fot.

Instruktionstips

Sätt dig på en mycket låg stol

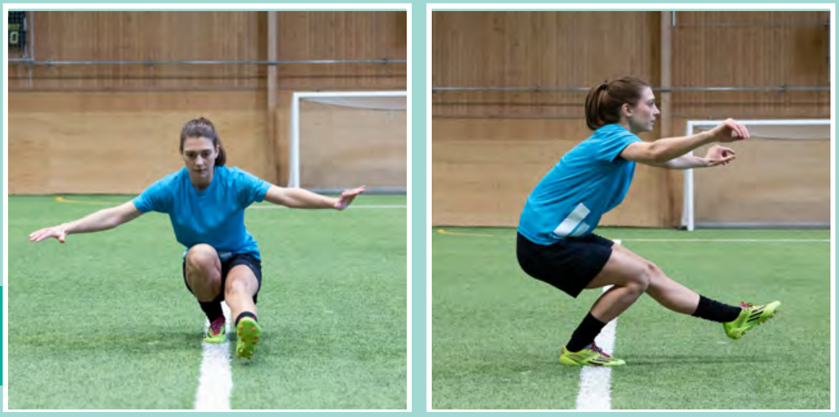

Parövningar

1G Enbensstående armdrag/knuff två och två.
Spelarna står på ett ben med lätt böjda knän och håller i varandras underarmar. Försök dra omkull varandra och samtidigt bibehålla en bra balans och knäet i samma riktning som foten. Alternativt försök knuffa varandra lätt på axeln. Byt ben efter 30 sekunder.

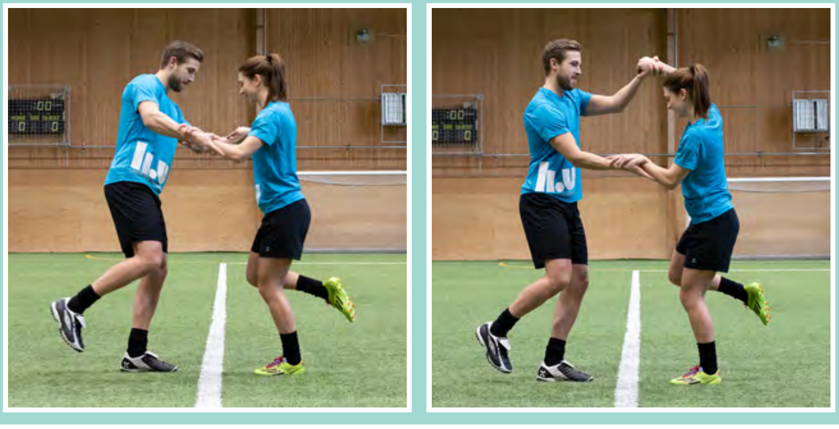

Enbensknäböj

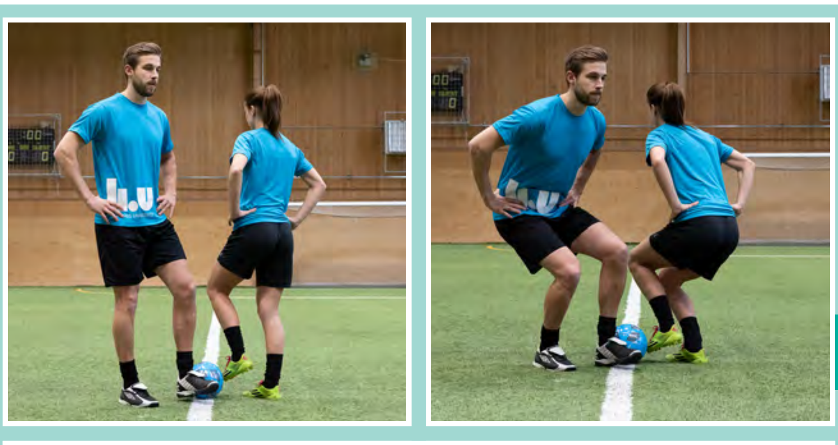

Parövningar

1H Fotpress mot boll.
Gör en enbensknäböj samtidigt som du pressar fria fotens utsida mot en boll. Den andra spelaren pressar samtidigt sin fria fot mot bollen. Om bollen hålls ovanför marken blir övningen svårare. Träna ett ben i taget.

Instruktionstips

Pressa foten mot bollen

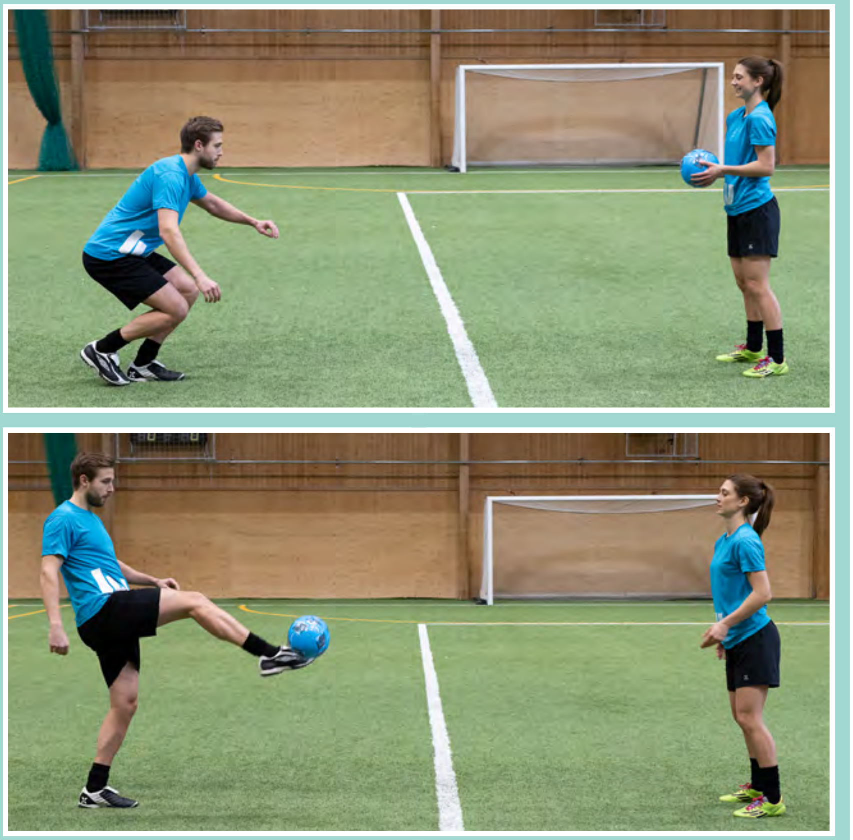

1I Enbensknäböj med spark.
Gör en enbensknäböj och sparka tillbaka bollen som en medspelare kastar till dig. Medspelaren kan utmana genom att kasta mot stödjebenet och att du svarar genom att snabbt byta stödjeben och sparka tillbaka bollen med andra foten.

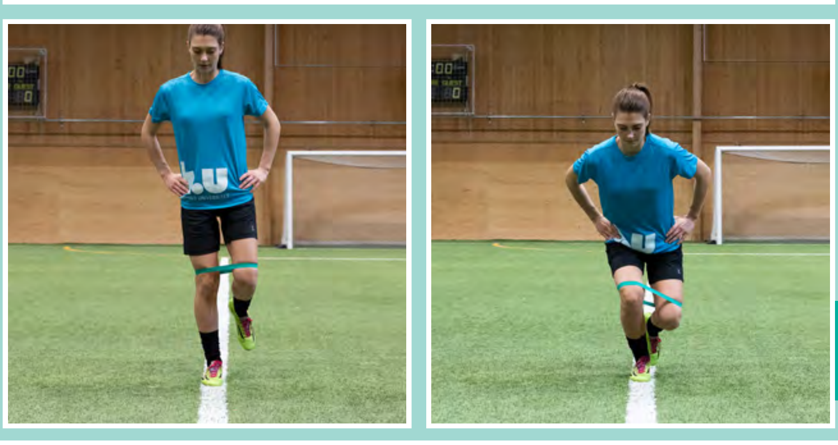

Gummibandsövning

1J Enbensknäböj med gummiband runt knäna.
Spänn ut benet för att sträcka upp gummibandet och gör enbensknäböj.

Instruktionstips

Håll gummibandet spänt hela tiden

2. Baksida lår (hamstrings)

Syftet är att stärka muskulatur i baksida lår, rumpa och bål. Fokusera på att spänna magmusklerna i samtliga övningar för att få stöd för ryggen och hålla ryggen rak. Gör övningarna långsamt och kontrollerat, 2 sekunder upp, 2 sekunder ner, om inget annat anges.

Övningar

2A Bäckenlyft med båda fötterna på marken.
Övningen blir lättare med fötterna nära rumpan och tyngre med fötterna långt ifrån.

Instruktionstips

Lyft omväxlande höfterna mot skyn / dutta rumpan i gräset

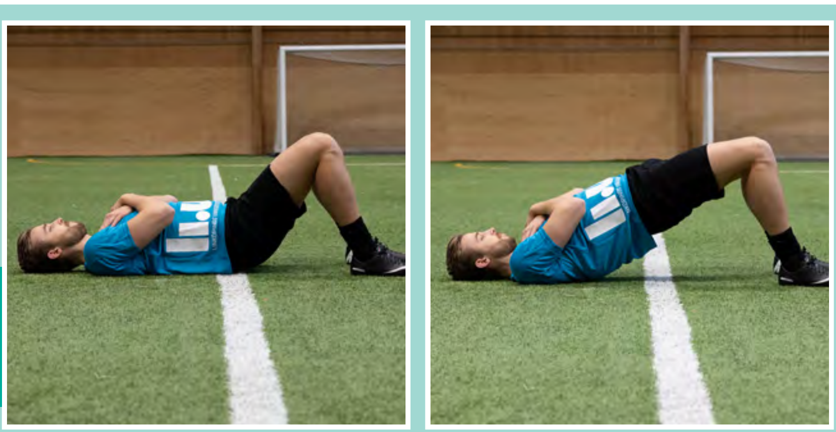

2B Bäckenlyft med boll mellan knäna.
Kläm åt bollen för att få extra träningseffekt på ljumskarna.

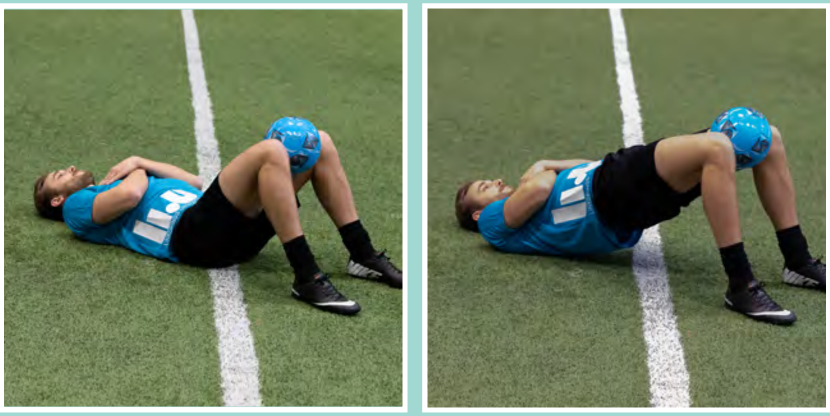

2C Bäckenlyft med en fot i marken.
Pressa ner ena foten i marken. Dra upp det andra benet mot magen och håll om det med båda händerna.

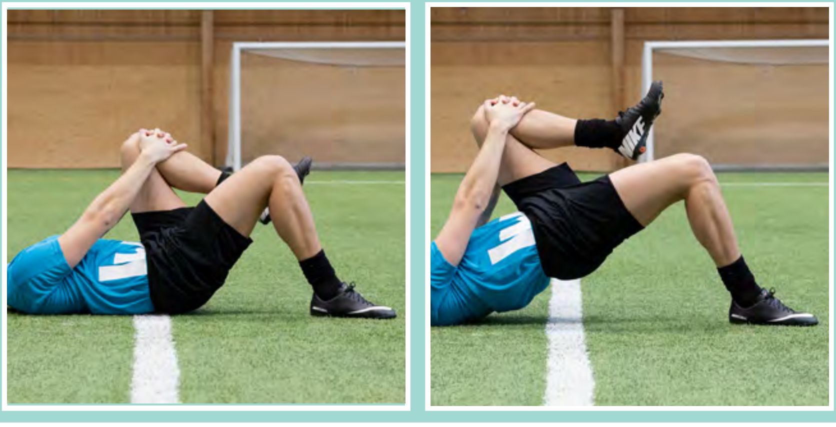

Baksida lår (hamstrings)

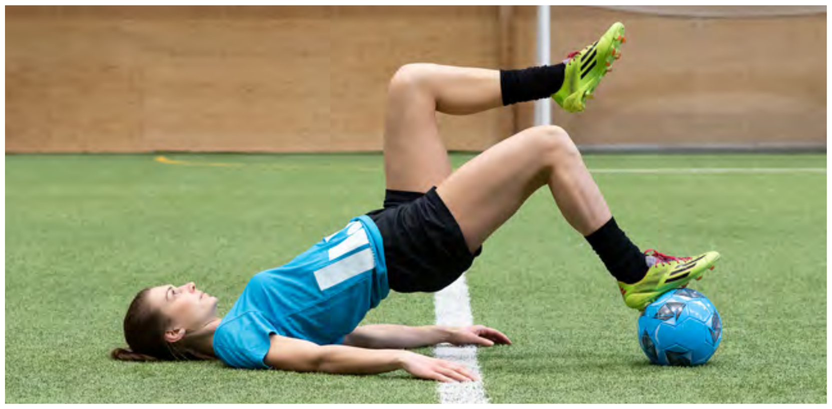

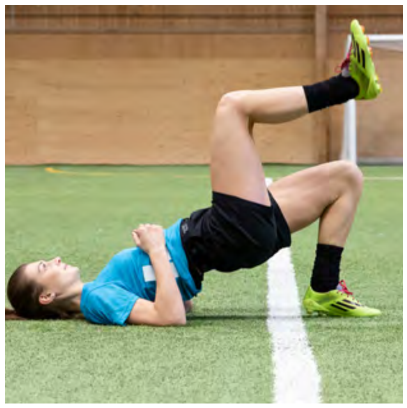

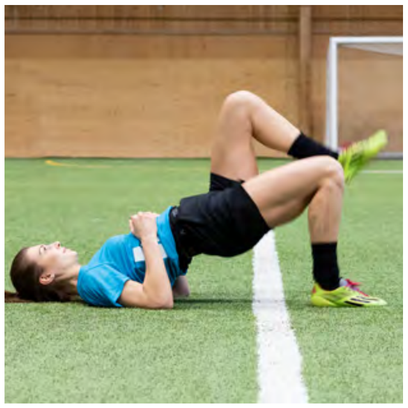

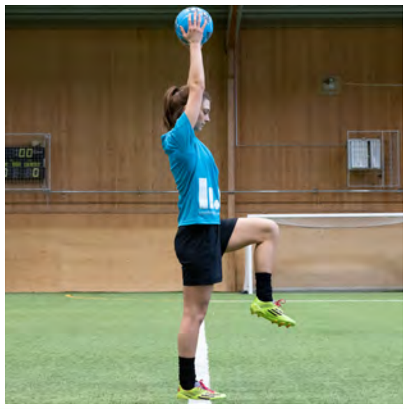

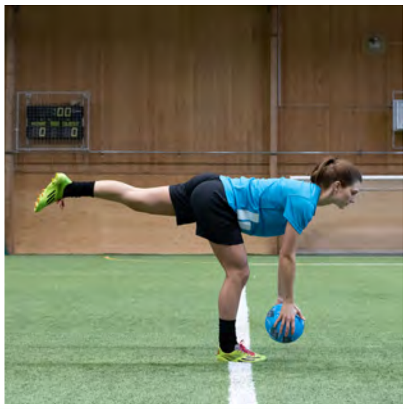

Övningar

2D Bäckenlyft med en fot på bollen.
Pressa fotsulan mot bollen. Dra upp det andra benet mot magen och ha armarna längs sidorna.

2E Bäckenlyft med explosivt frånskjut.
Ligg med ena foten i marken och den andra i luften. Ha armarna längs med sidorna med 90 graders vinkel i armbågarna. Lyft rumpan och behåll i samma position hela övningen. Skjut ifrån med den fot som har markkontakt och växla snabbt fot i marken genom att landa på motsatt fot

2F Draken.
Stå med lätt böjt knä. Fäll i höften på ståbenet, sträck fria benet rakt bak och doppa bollen mot gräset. Tips – vrid in tårna på fria benet så är det lättare att hitta rätt teknik. Räta upp kroppen och sträck bollen över huvudet. Behåll höfterna parallella. Träna samma ben i 30 sekunder. Obs – tröttheten ska kännas i baksida lår på stödjebenet!

Baksida lår (hamstrings)

Parövningar

2G Enbensbäckenlyft med ena benet upplyft.
Ligg på marken med händerna i kors på bröstet. Din medspelare står med böjda knän och håller runt hälen på din ena fot.

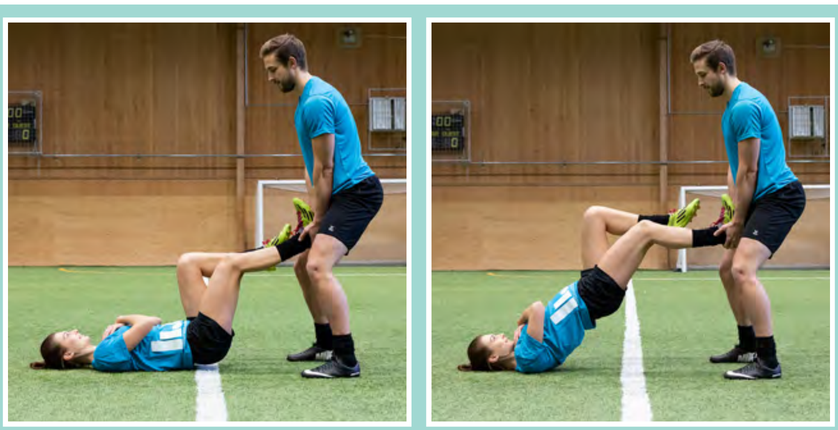

2H Nordic hamstrings.
Stå på knä medan en medspelare fixerar dina ben. Luta dig långsamt framåt så långt det går med god hållning. När det inte går att hålla emot längre går du ner i en armhävning och trycker dig upp till utgångspositionen med hjälp av armarna. Denna övningsvariant kan vara mycket belastande och bör göras max två gånger per vecka under försäsong, en gång per vecka under tävlingssäsong.

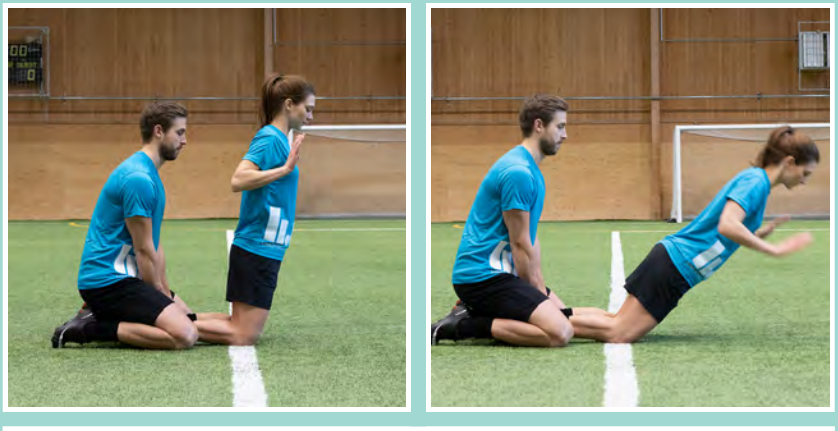

Gummibandsövningar

2I Bäckenlyft med gummiband.
Gummiband runt knäna, spänn upp gummibandet genom att pressa isär knäna.

Instruktionstips

Håll gummibandet sträckt

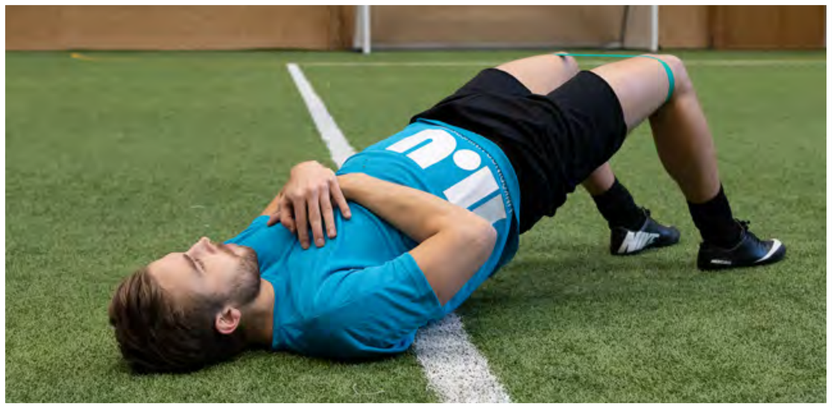

2J Stående hamstringscurl.
Gummiband vid vristerna. För foten snabbt mot rumpan. Träna en sida i taget ca 30 sekunder.

Instruktionstips

Sparka dig snabbt i rumpan

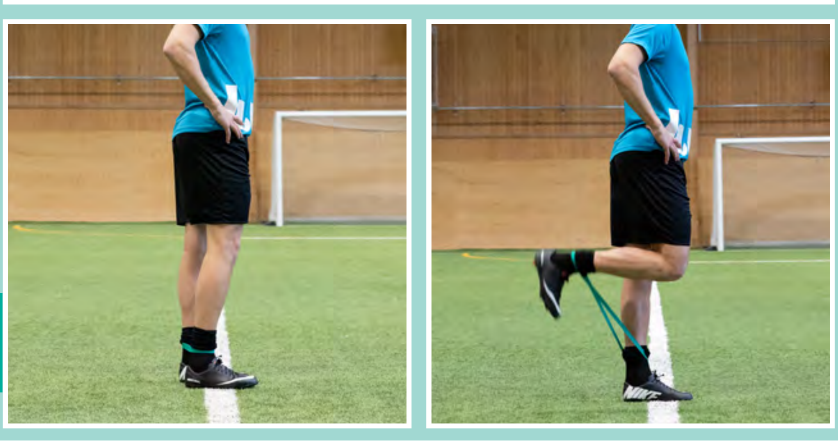

Knäböj på två ben

3. Knäböj på två ben

Syftet är att träna styrka och rörlighet över flera leder. Fokusera på att göra en rörelse som att sätta dig på en så låg stol som möjligt med knän och fötter pekandes rakt fram, hela fotsulorna i marken och stabil bål. Spänn magmusklerna för att få stöd för ryggen. Gör en långsam rörelse, ca 2 sekunder ner, 2 sekunder upp.

Instruktionstips

Sätt dig på låg en stol

Övningar

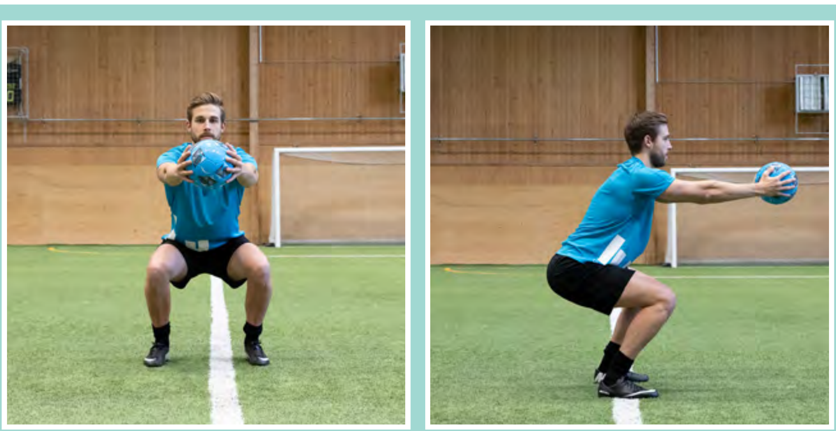

3A Knäböj med armarna framåt, håll i en boll.
Gör knäböj i lugnt tempo

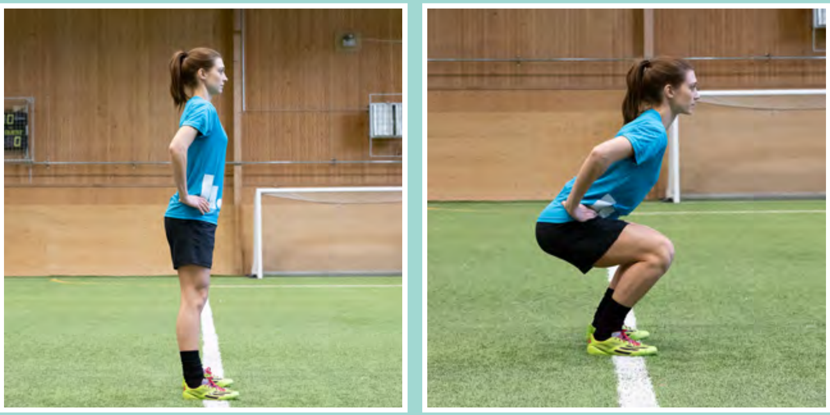

3B Knäböj med händerna vid höfterna.
Gör knäböj i lugnt tempo

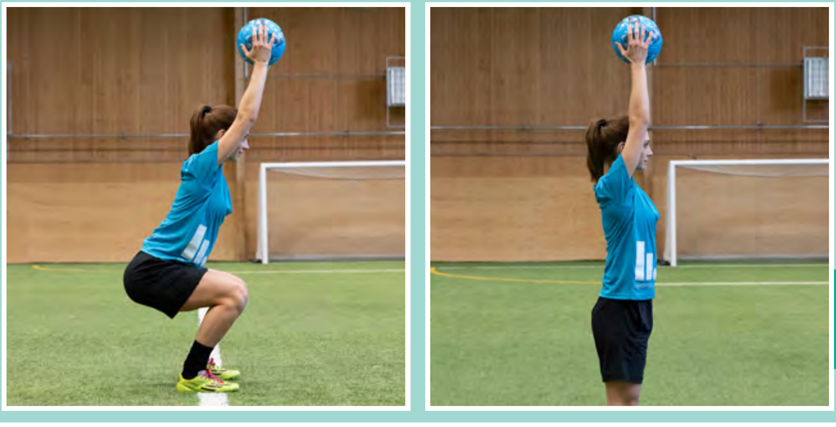

3C Knäböj med raka armar över huvudet.
Sträck bollen högt upp i skyn medan du gör knäböj.

Instruktionstips

Sträck bollen upp mot skyn

Knäböj på två ben

Övningar

3D Knäböj med raka armar över huvudet, upp på tå.
Gör en knäböj, sträck sedan på dig och gå upp på tå.

Instruktionstips

Sträck bollen upp mot skyn

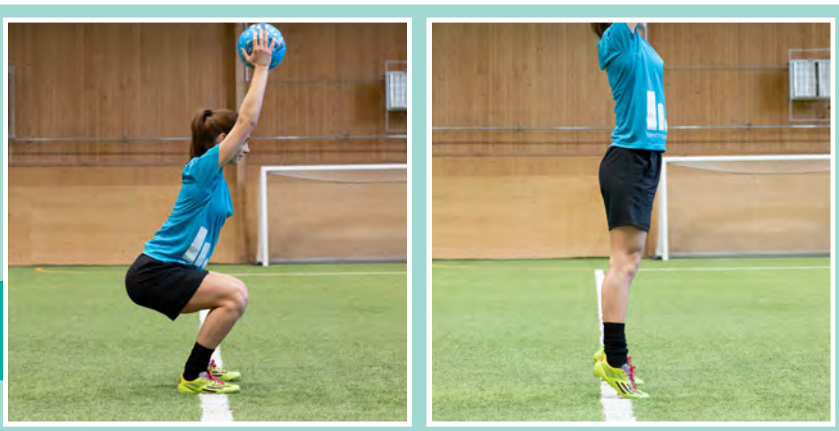

3E Djupgång i sidled.
Stå som om du satt i en stol, gå i sidled med benen böjda, en riktning i taget.

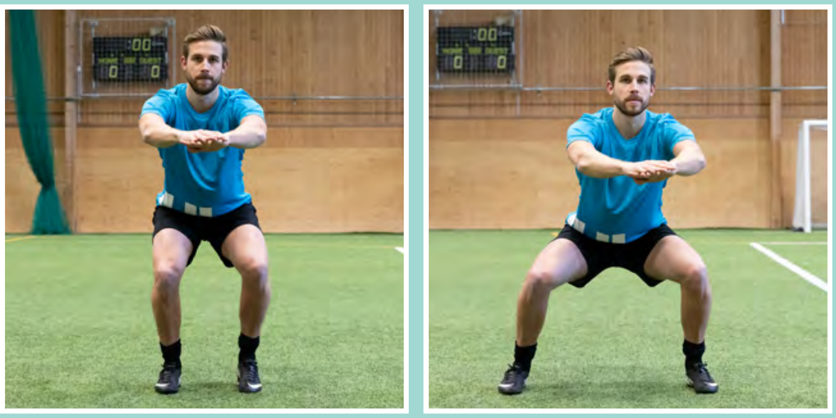

3F Djupa hopp.
Hoppa framåt, landa mjukt och böj på knäna.

Instruktionstips

Landa mjukt

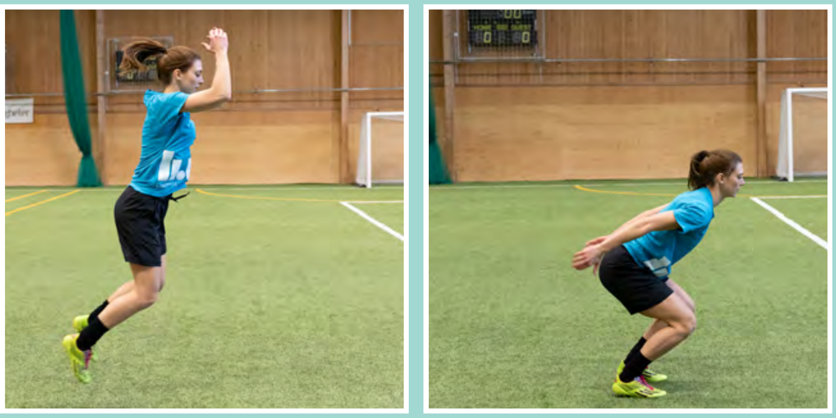

Parövningar

3G Knäböj med bollstöd.
Gör en rörelse som att sätta dig på en låg stol samtidigt som du och en medspelare håller bollen mellan er med varsin hand.

Instruktionstips

Sätt er på varsin låg stol

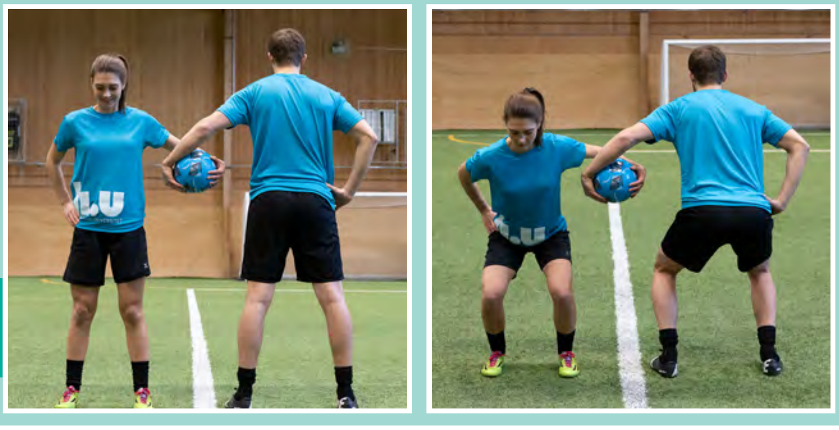

Knäböj på två ben

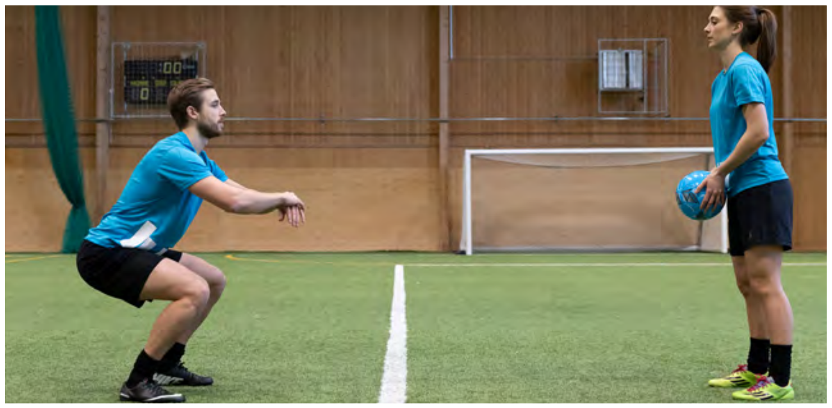

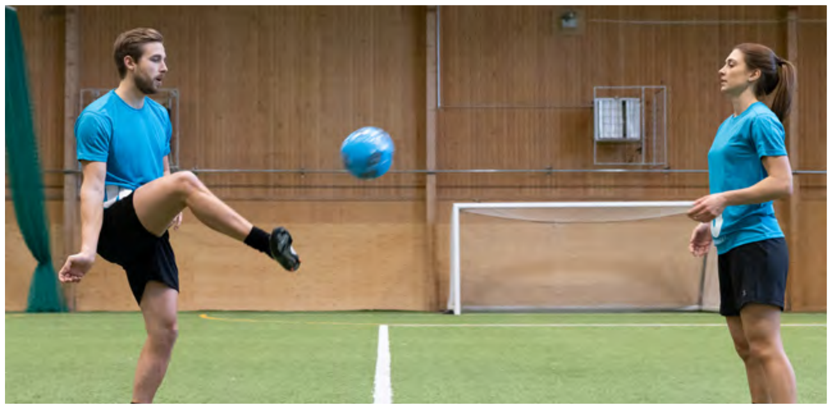

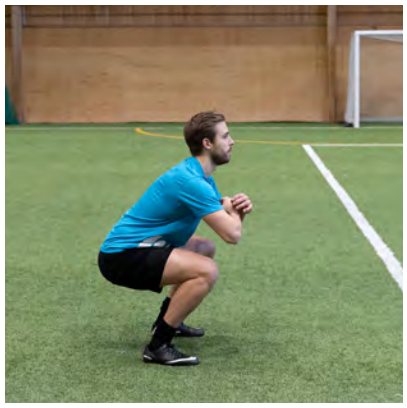

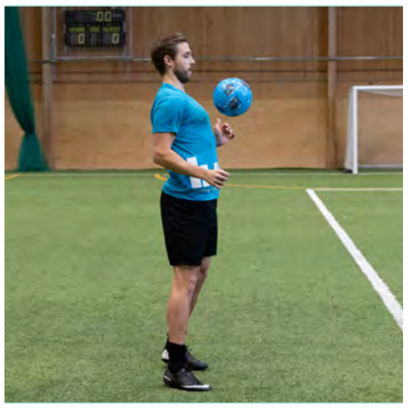

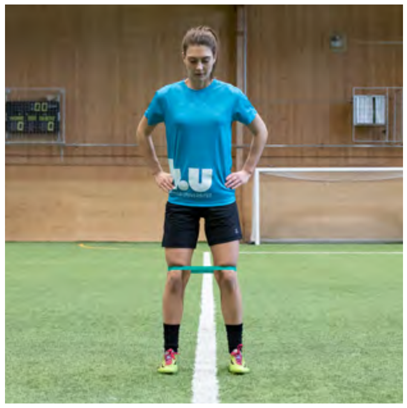

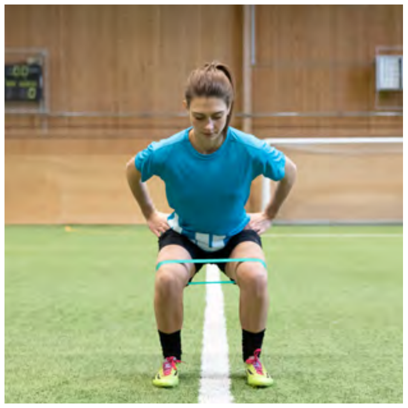

Parövningar

3H Knäböj med benuppdrag och bollkick. Gör en knäböj. I samband med att du sträcker på benen kastar medspelaren en boll mot dig. Sparka tillbaka bollen på volley.

Instruktionstips

Sätt dig på låg en stol

3I Knäböj med bollmottagning. Gör knäböj, medspelaren kastar en boll mot dig. Ta ner bollen på bröstet och sparka tillbaka.

Instruktionstips

Ta ner bollen på bröstet medan du sträcker på dig

Gummibandsövning

3J Knäböj med gummiband runt knäna. Gör en rörelse som att sätta dig på en låg stol, pressa samtidigt isär knäna och spänn upp gummibandet.

Instruktionstips

Håll gummibandet spänt hela tiden

4. Bålstyrka

Syftet är att träna bålstyrka och kontroll. Fokusera på att spänna mag- och ryggmuskler i samtliga övningar. Bibehåll neutral svank och en god hållning genom hela övningen. Försök hålla magen platt. Tröttheten ska kännas i magen, inte i ryggen. Träna i 30-60 sekunder.

Övningar

4A Planka med knäna i marken.
Stöd på underarmarna med armbågarna rakt under axlarna. Spänn mage och rumpa.

Instruktionstips

Stå rak och stilla som en planka

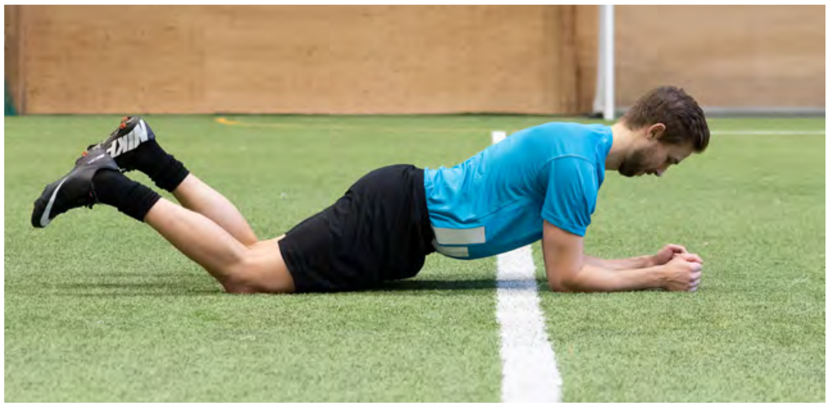

4B Planka med tårna i marken.
Stöd på underarmarna med armbågarna rakt under axlarna. Spänn mage och rumpa.

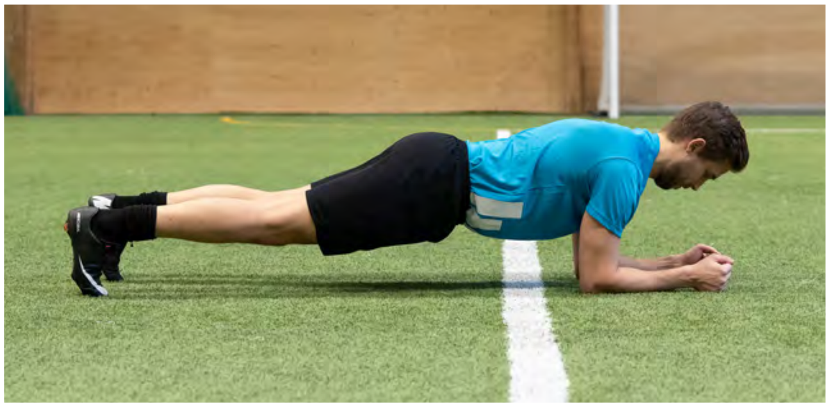

4C Sidplanka stillastående.
Stå med ena underarmen och foten i marken, håll stilla.

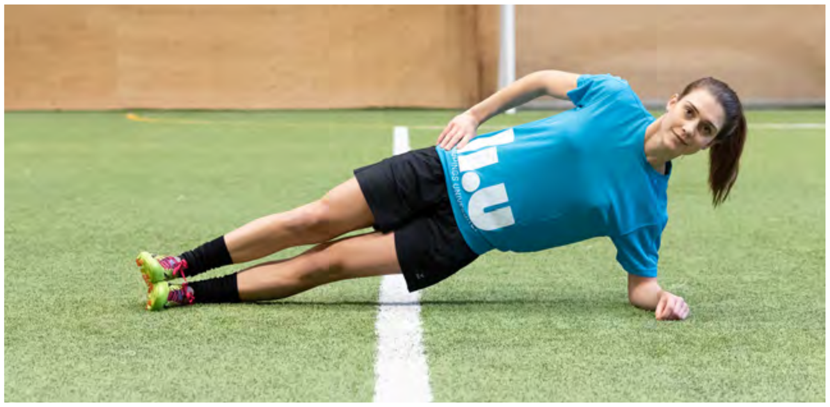

Övningar

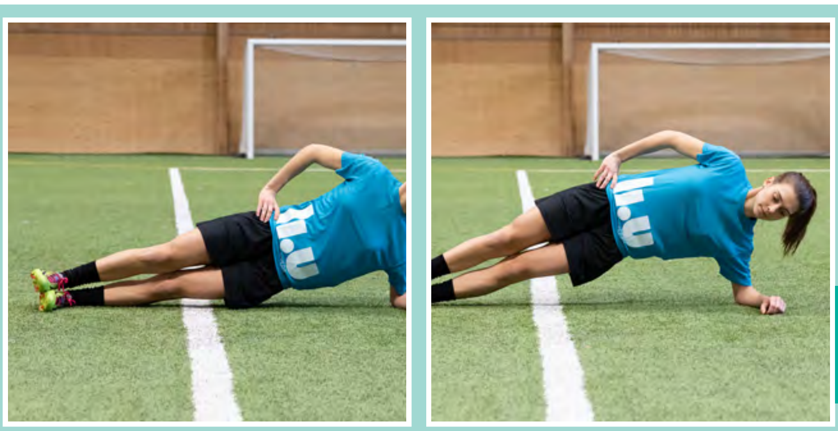

4D Sidliggande höftlyft.
Ligg på sidan med tyngden på underarmen och ena foten. Håll armbågen rakt under axeln. Spänn mage och rumpa.

Instruktionstips

Lyft omväxlande höften mot skyn / dutta mot gräset

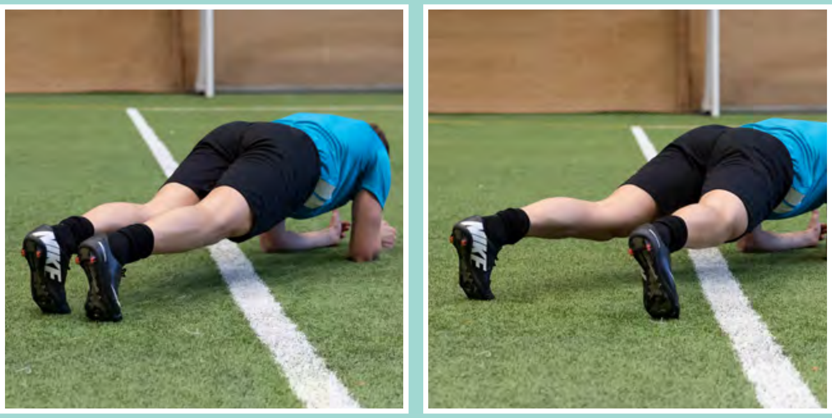

4E Planka på tå med fotförflyttning.
Stöd på underarmarna och spänn mage och rumpa. Flytta båda fötterna växelvis utåt och inåt.

Instruktionstips

Håll stilla höfterna

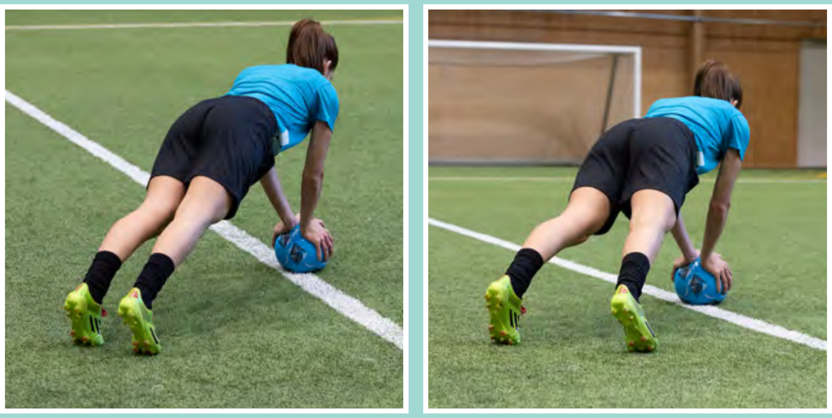

4F Hög planka på boll med fotförflyttning.
Stå med händerna på en boll, samtidigt som du flyttar fötterna växelvis utåt och inåt.

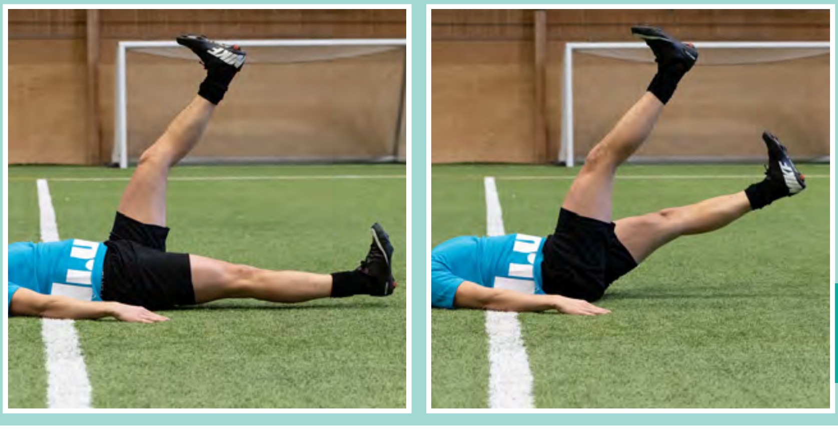

4G Benfällning.
Ligg på rygg och pressa svanken mot marken. Dutta omväxlande en häl i taget i gräset. Ju mer benet sträcks, desto tyngre.

Instruktionstips

Pressa svanken mot gräset

Parövningar

4H Sit-up med boll.
Ligg med böjda ben mittemot varandra och fotsulorna mot varandra. Gör en sit-up och kasta bollen till en medspelare som gör sit-ups i samma tempo som dig .

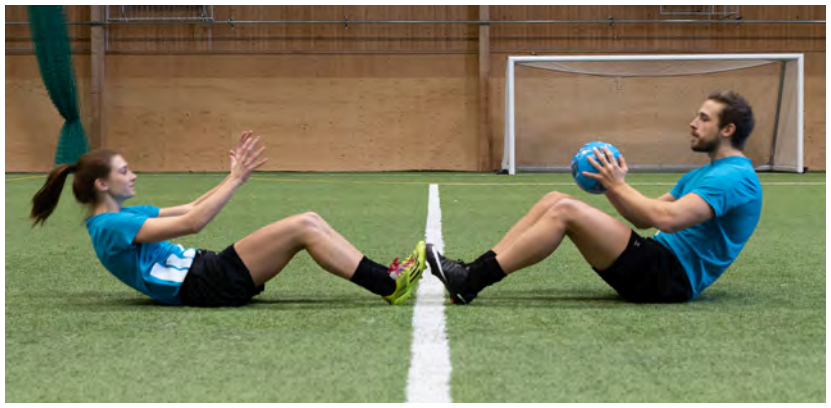

4I Hög planka med handklapp.
Stå som en planka med händer och fötter i gräset. Klappa en hand i taget mot medspelarens hand.

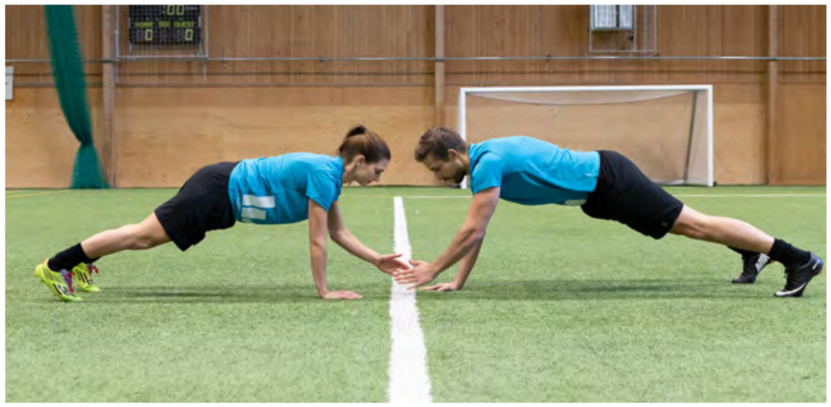

4J Skottkärran.
Stå som en planka med händer i gräset medan en medspelare håller i dina fötter. ”Gå” en sträcka framåt med händerna medan medspelaren lyfter benen.

Instruktionstips

Gå en sträcka framåt. Håll bålen rak och stilla som en planka

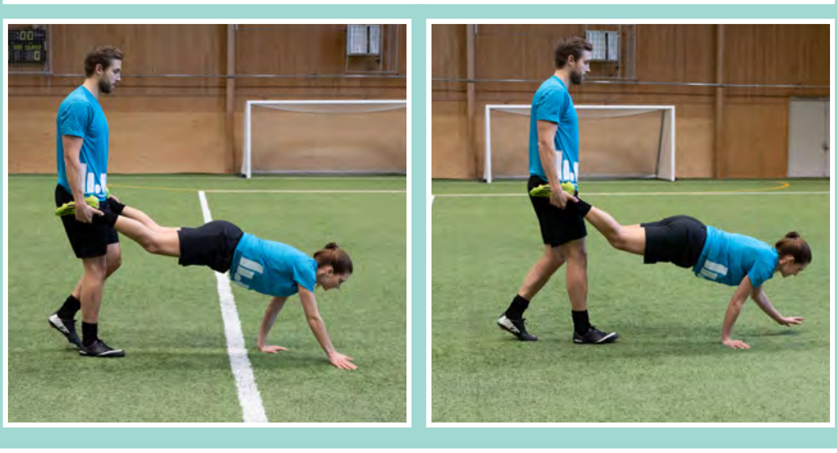

5. Utfall

Syftet är att träna knäkontroll och styrka i bål och ben. Fokusera på att göra utfallssteg med fötter och knän pekande i samma riktning och att vända mjukt i rörelsen. Tänk att kroppen ska höjas och sänkas rakt upp och ner som en hiss. Spänn mage och rumpa för god bålkontroll. Undvik att tappa ner rygg eller höft åt sidan eller att sätta ner bakre knäet i marken. Gör en långsam rörelse, ca 2 sekunder ner, 2 sekunder upp.

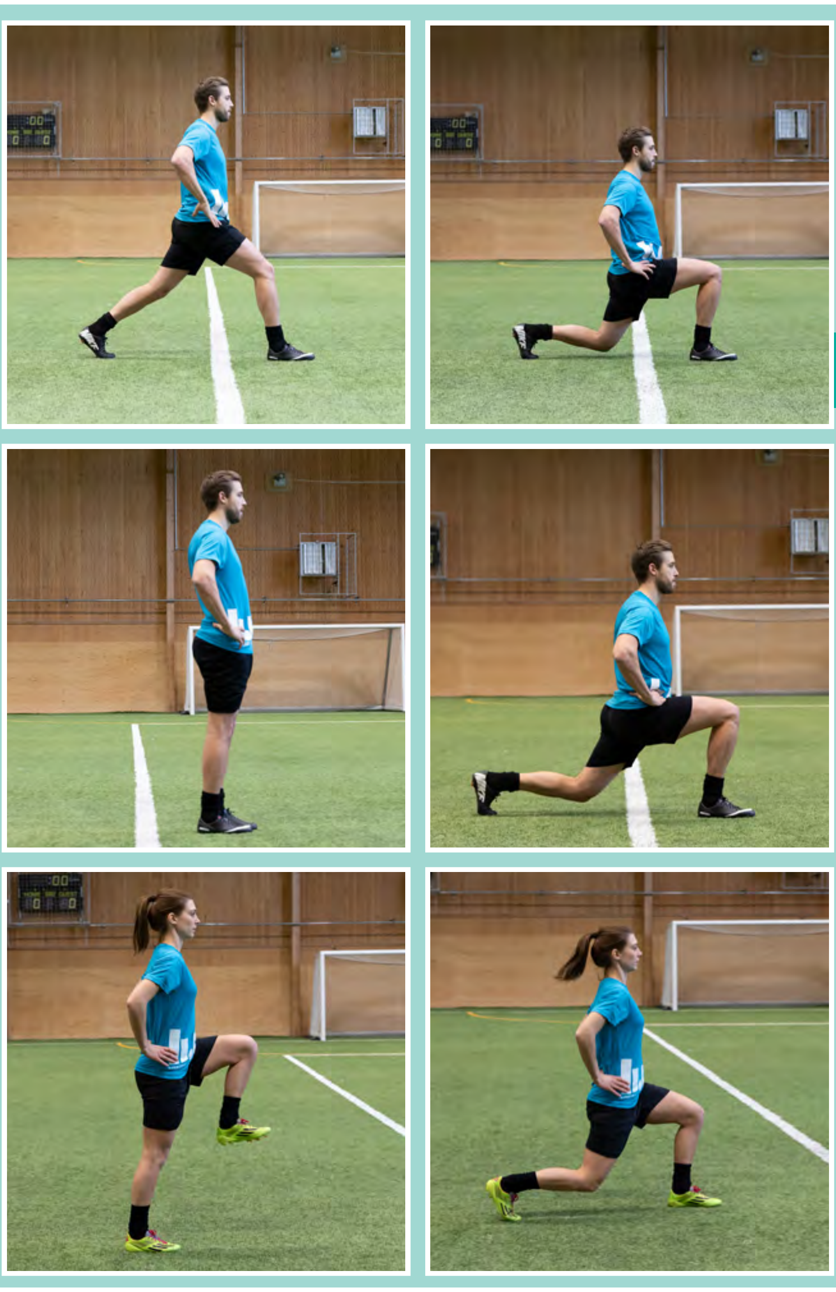

Övningar

5A Stillastående utfallssteg.
Stå i ett utfallssteg med höftbredd mellan fötterna och med foten så långt fram att det blir en rät vinkel i främre knäet. Kroppstyngden är lika mycket på båda benen. Höj och sänk kroppen rakt upp, rakt ner.

Instruktionstips

Rör dig som en hiss

5B Utfallssteg bakåt.
Ta ett stort kliv bakåt och sjunk ner i ett utfallssteg, gå tillbaka till utgångspositionen och byt ben.

5C Utfallsgång framåt.
Gå med stora, djupa kliv framåt. Lyft först knäet högt mot skyn och sjunk sedan ner djupt i ett utfall. Sätt foten tyst i marken. Gå även upp på tå om du orkar och har balans.

Övningar

5D Utfallsgång framåt med rotation.
Gå med stora, djupa kliv framåt. För samtidigt bollen från sida till sida. När vänster ben är fram ska du vrida bålen åt vänster, när höger ben är fram ska du vrida bålen åt höger.

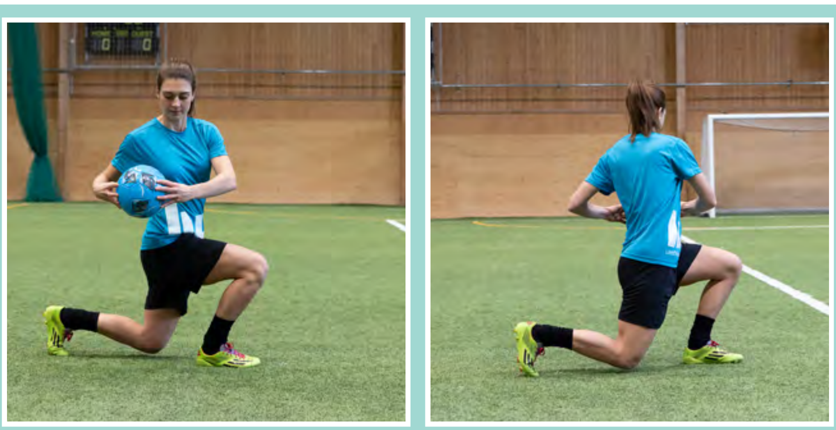

5E Utfallssteg på stället med raka armar.
Ta ett stort kliv framåt, lyft först knäet högt mot skyn och sjunk sedan ner djupt i ett utfall. Tryck dig tillbaka med hjälp av främre foten. Håll bollen högt i skyn hela tiden.

Instruktionstips

Sätt foten mjukt i marken

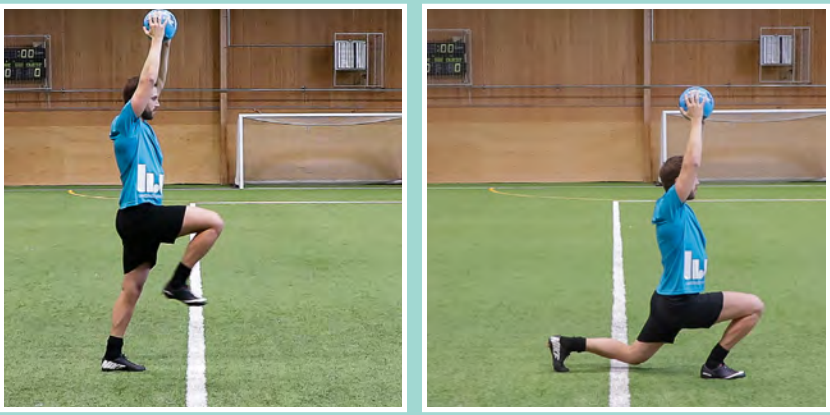

5F Utfallssteg i sidled.
Stå med fötterna axelbrett och håll en boll framför kroppen med raka armar. Gör ett utfallssteg i sidled och flytta tyngden till det yttre benet och sträck samtidigt det andra benet rakt i sidled. Tryck dig tillbaka till utgångspositionen.

Instruktionstips

Sätt foten mjukt i marken

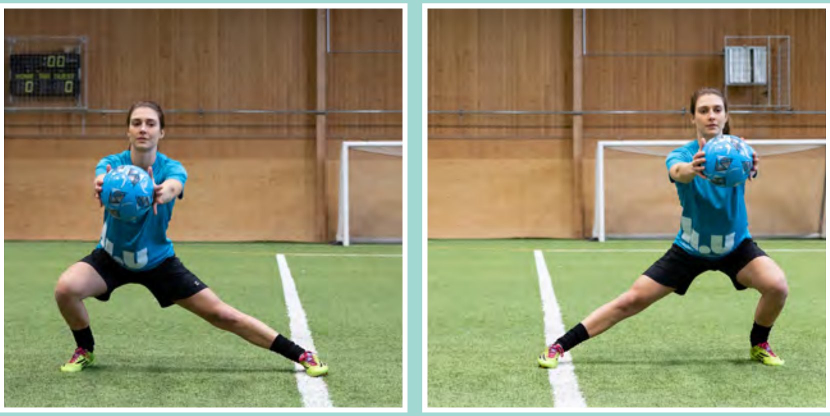

5G Utfallshopp.
Stå i ett djupt utfallssteg, hoppa och byt snabbt plats på fötterna som en skidåkare.

Instruktionstips

Landa fjäderlätt

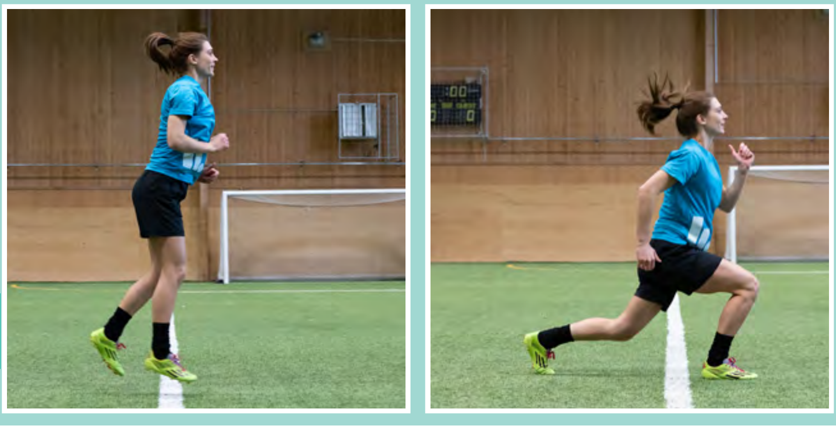

Utfall

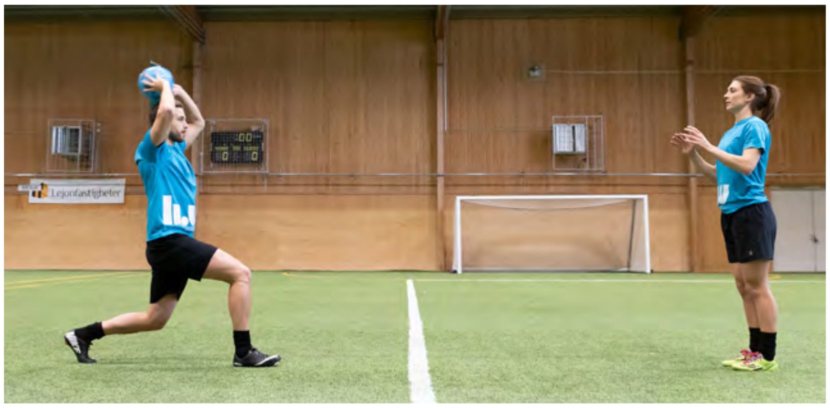

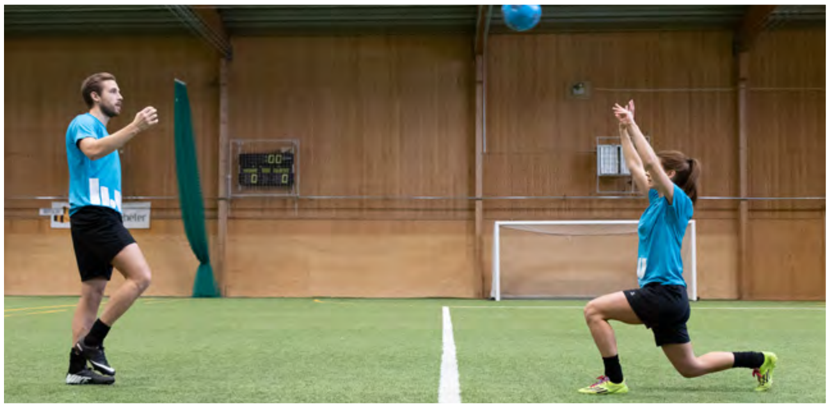

Parövningar

5H Utfallssteg med inkast.
Gör ett inkast till en medspelare som står mittemot och kliv samtidigt framåt i ett utfallssteg med god bålkontroll. Gör ett markerat knälyft följt av en mjuk, dämpad landning. Vänd direkt tillbaka till utgångsposition och ta emot.

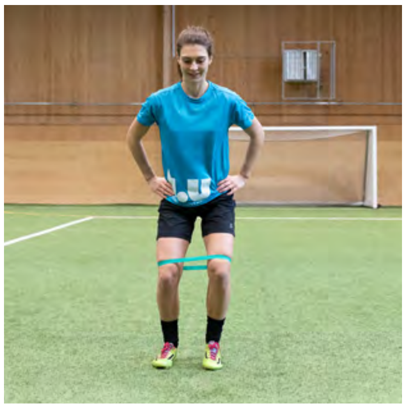

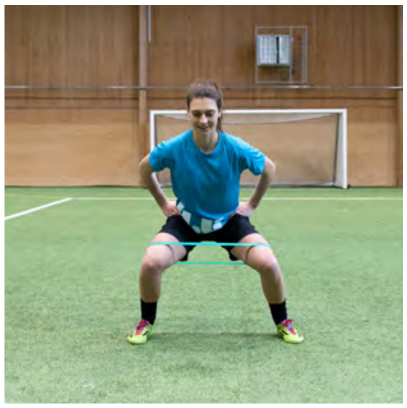

Gummibandsövning

5I Utfallsgång i sidled.
Kort hävarm. Gummiband runt knäna, böj på benen och kliv åt sidan med god knäkontroll.

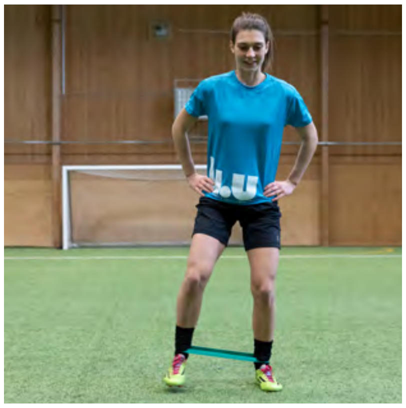

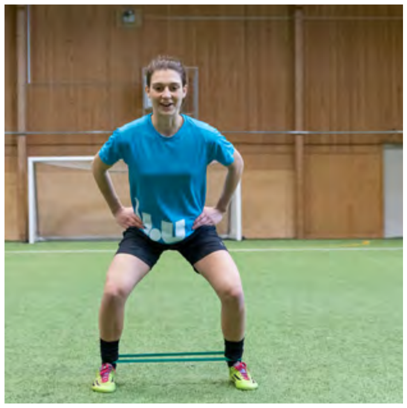

5J Utfallsgång i sidled.
Lång hävarm (tyngre). Gummiband runt fotleder, böj på benen och kliv åt sidan med god knäkontroll.

6. Hopp/landning

Syftet är att träna på mjuka, dämpade landningar med bål- och knäkontroll. Fokusera på att landa mjukt och ljudlöst med knän och höfter böjda och med god kontroll på bålen. Stanna upp för att återfå balansen innan nästa hopp påbörjas. Var noga med att knä och fot pekar i samma riktning.

Instruktionstips

Landa mjukt

Övningar

6A Tvåbenshopp.
Hoppa jämfota framåt/bakåt/till sidan (ska kunna göras med god teknik innan enbenshopp introduceras). Fötter och knän pekar rakt fram.

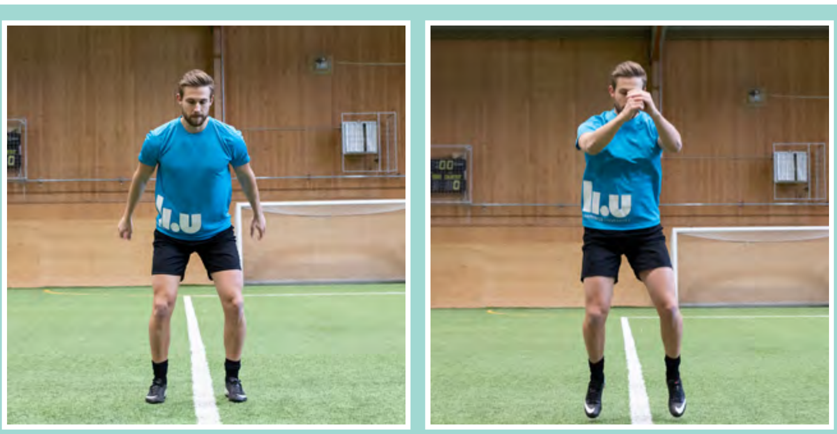

6B Skridskohopp i sidled.
Hoppa skridskohopp från sida till sida. Landa mjukt och med god kontroll över bål och ben.

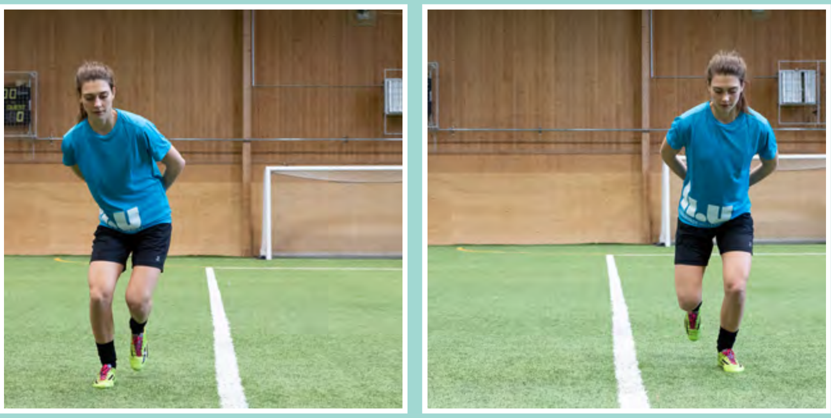

6C Enbenshopp.
Gör ett hopp framåt med kontroll i landningen, frys en kort stund, hoppa sedan tillbaka till utgångspositionen med kontroll.

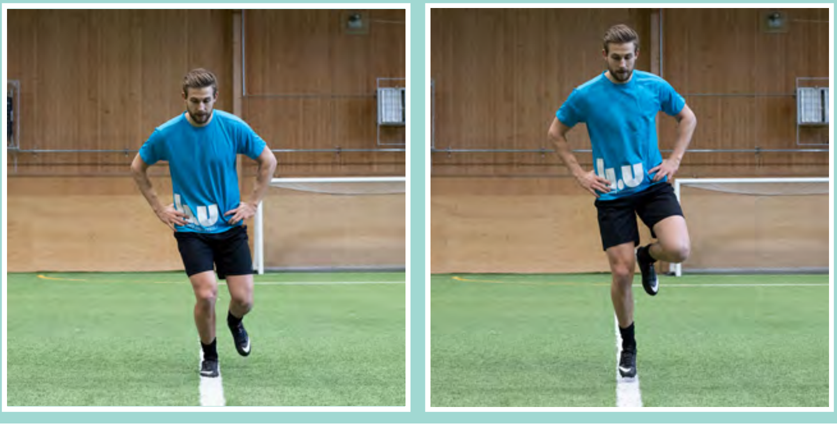

Hopp/landning

Övningar

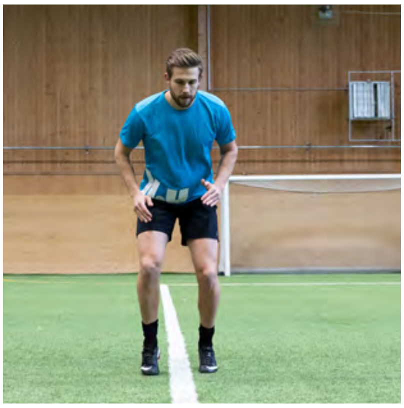
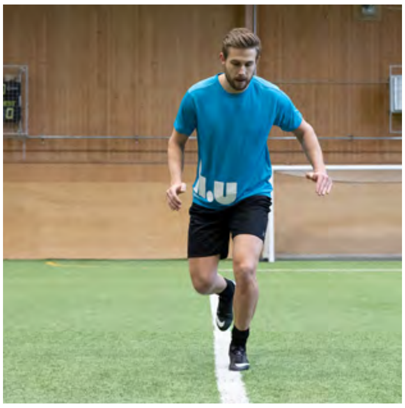

6D Utfallshopp framåt.
Jogga med snabba fötter på stället och gör ett utfallshopp framåt. Landa mjukt och frys landningen en kort stund. Tips: tränaren kan använda visselpipa och styra hoppen.

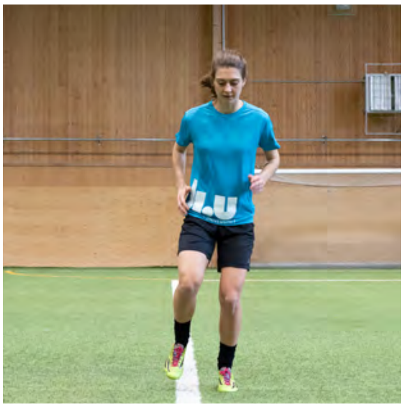
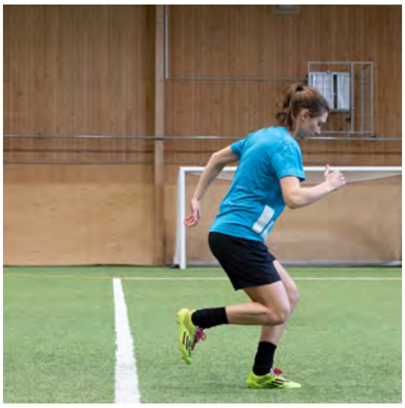

6E Riktningsförändring.
Jogga med snabba fötter på stället och hoppa 90 grader åt ena sidan. Frys landningen och stå kvar en kort stund. Växla sida. Tips: tränaren kan ropa åt vilket håll spelarna ska vrida sig.

Instruktionstips

Snabba fötter

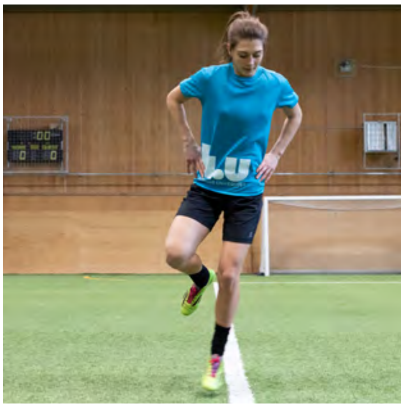
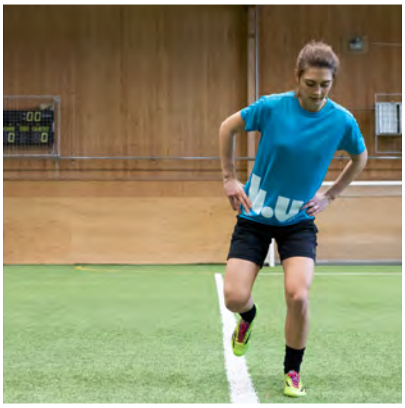

6F Enbenshopp i kvadrat.
Stå på ett ben och hoppa åt höger, vänster, framåt, bakåt i form av en kvadrat.

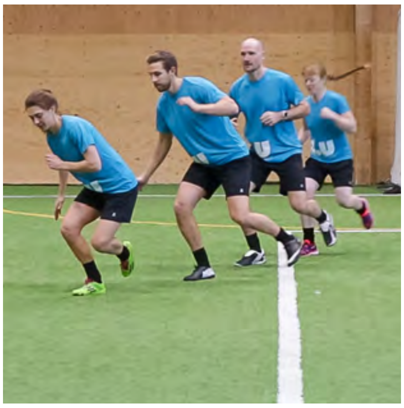
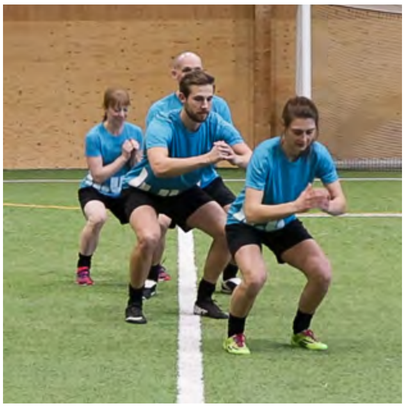

Parövningar

6G Följa John-hopp med förflyttning.
Spelaren längst fram bestämmer vad det blir för hopp, medspelarna härmar. Kan göras i olika riktningar samt med tvåbens- eller enbenslandning.

Parövningar

6H Spring huller-om-buller med handklapp/axelknuff.
Alla springer oorganiserat och gör upphopp med handklapp/axelknuff med närmaste medspelaren och landar på båda benen innan de fortsätter springa.

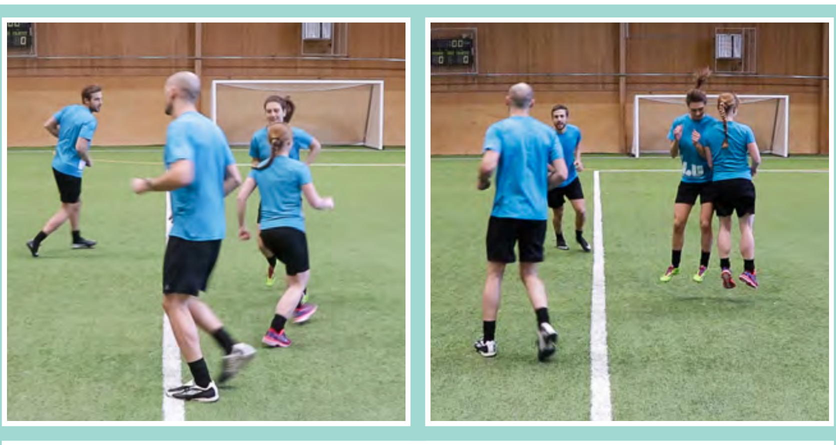

6I Upphopp med nick.
Gör ett upphopp och nicka en boll som en medspelare kastar. Landa på båda fötterna med god knäkontroll och lätt böjda knän. Frys landningen och stå kvar en kort stund.

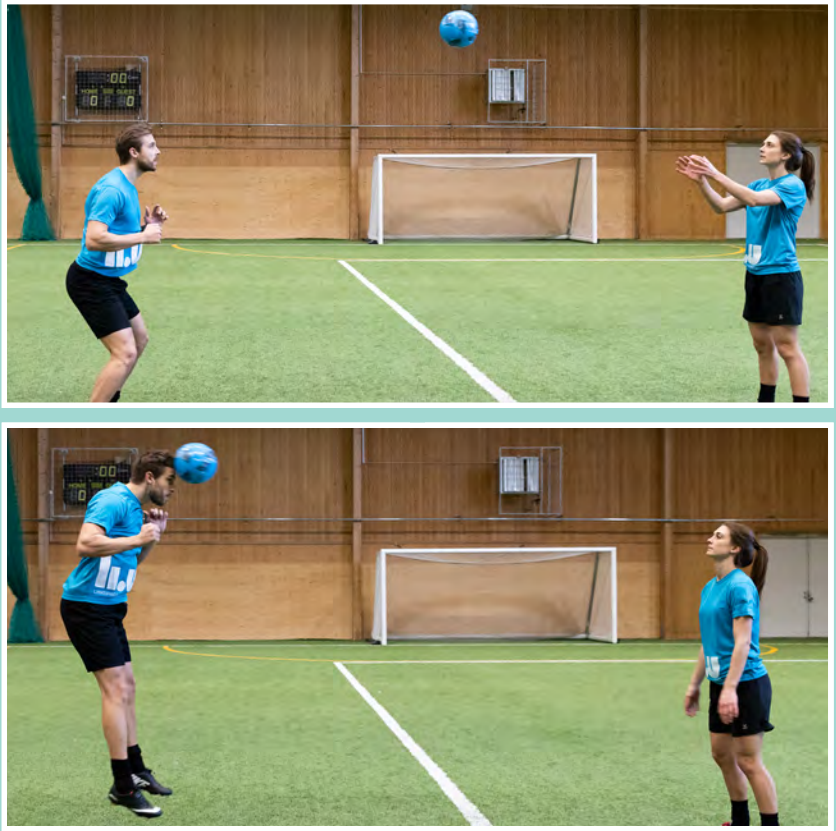

6J Upphopp med knuff.
Gör ett upphopp och landa med knäkontroll på två eller ett ben samtidigt som en medspelare knuffar dig i olika riktningar under hoppet.

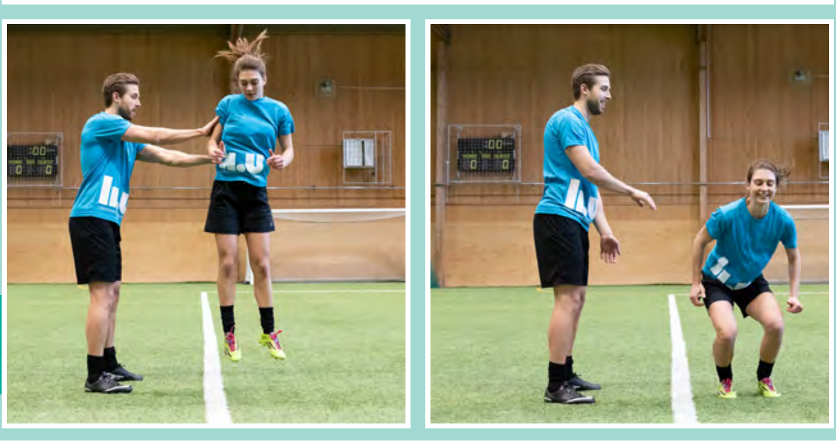

Instruktionstips

Landa mjukt, hitta balansen

Anteckningar

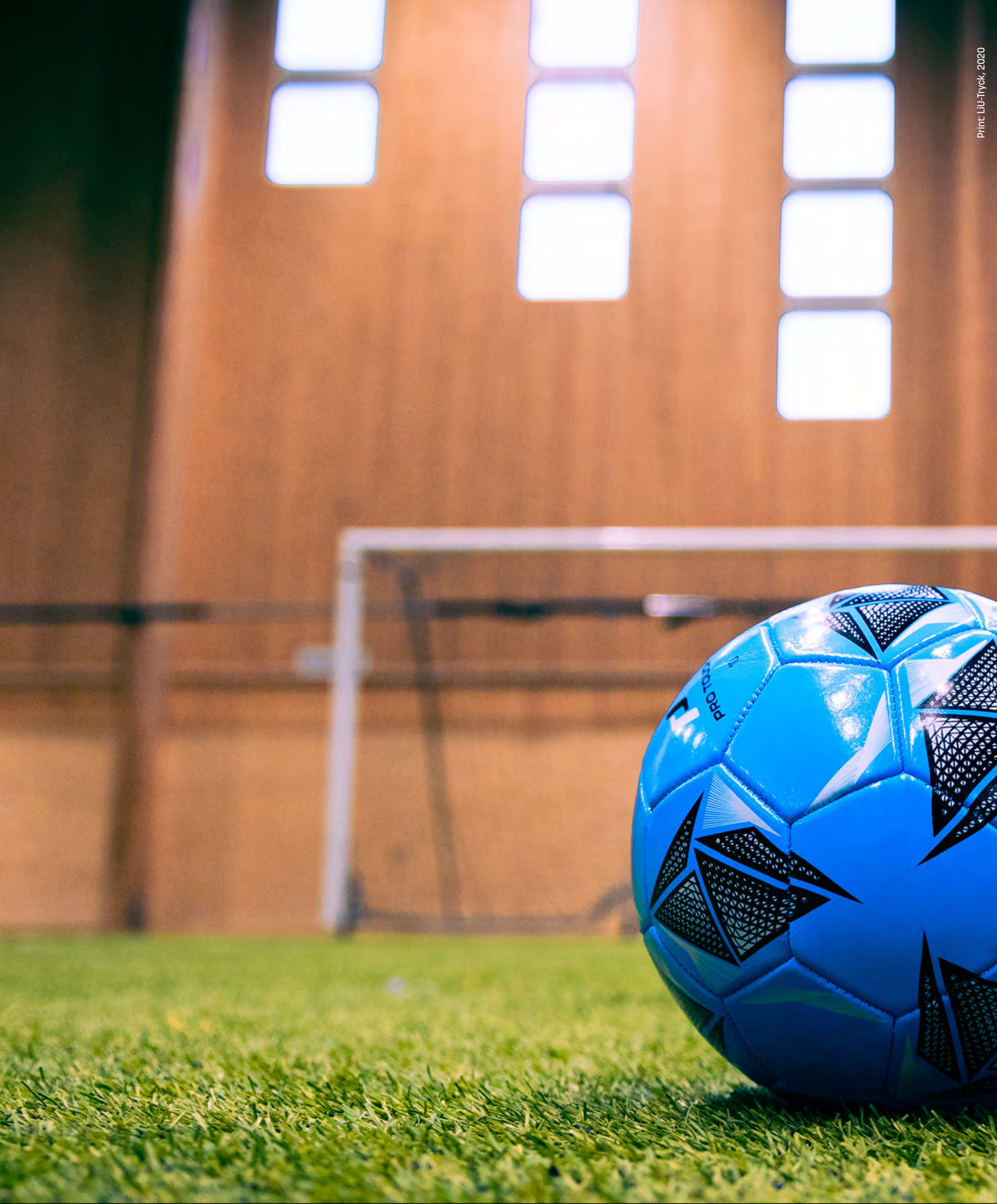

Print: LiU-Tryck, 2020

LINKÖPINGS UNIVERSITET

SWIPE Sport Without Injury ProgrammE
