# Supplementary material for: Adherence to Injury Prevention Exercise Programmes in Amateur Adolescent and Adult Football: A Detailed Description of Programme Use from a Randomised Study
Source: Sports Med Open. 2023 Jul 15;9:57. doi: 10.1186/s40798-023-00608-1 (PMC10349794; doi:10.1186/s40798-023-00608-1)
Supplement: Supplementary file 2 — Additional file 2. Adductor strength programme, original version and Adductor strength programme, alternative exercises that were added due to the pandemic [file 40798_2023_608_MOESM2_ESM.pdf]

Sports Medicine – Open. Adherence to injury prevention exercise programmes in amateur adolescent and adult football— A detailed description of programme use from a randomised study. Lindblom H, Waldén M, Hägglund M.

Affiliation for corresponding author: Unit of Physiotherapy, Department of Health, Medicine and Caring Sciences, Linköping University, Linköping, Sweden. Sport Without Injury ProgrammE (SWIPE), Department of Health, Medicine and Caring Sciences, Linköping University, Linköping, Sweden
E-mail address for corresponding author: hanna.lindblom@liu.se

Hanna Lindblom, Markus Waldén and Martin Hägglund declare that they have no competing interests.

The study was funded by grants from the Swedish Research Council Ref. no. 2018-03135 and Region Östergötland Ref. No. 922771.

Styrketräningsprogram för ljumskmuskulaturen

Upplägg

Uppvärmning
Vi rekommenderar att spelarna värmer upp innan träningsprogrammet.

Styrketräning för ljumskmuskulaturen (**insida lår**)
Programmet genomförs 2-3 gånger per vecka under försäsong, och 1 gång per vecka under tävlingssäsong. Träna ett set per ben per träningstillfälle. Övningen finns på tre nivåer, där 1 är lättast och 3 är svårast. Börja med övningsnivå 3 för alla spelare.

- Välj en lättare övningsnivå om spelaren inte klarar att utföra övningen med korrekt teknik och avsett antal repetitioner, se tabell över träningsdosering nedan.
- Välj en lättare övningsnivå om spelaren får ont vid utförande av övningen motsvarande >3 på en skala från 0-10 (där 0 är ingen smärta och 10 är värsta tänkbara smärta), se figur nedan.

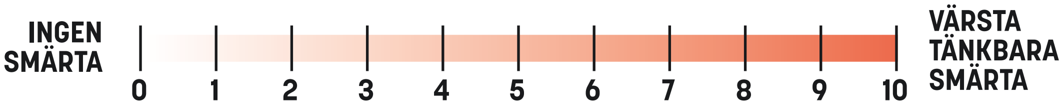

Smärtskala.
Välj en lättare övningsnivå om spelarna upplever en smärta >3 i samband med övningen.

Träningsdosering

Följ schemat from v 1 under försäsongen så långt ni hinner. Påbörja underhållsträning 1 pass per vecka i samband med den första seriematchen och fortsätt med samma dosering hela säsongen ut.

Uppbyggnad försäsong (vecka)	Antal träningspass/ vecka	Antal set/ ben	Antal repetitioner/ ben
1	2	1	3-5
2	3	1	3-5
3-4	3	1	7-10
5-6	3	1	12-15
7-8	2	1	12-15
**Underhåll tävlingssäsong**	1	1	12-15

SWIPE Sport Without Injury ProgrammE • Studieansvariga: Hanna Lindblom, Martin Hägglund

LINKÖPINGS UNIVERSITET

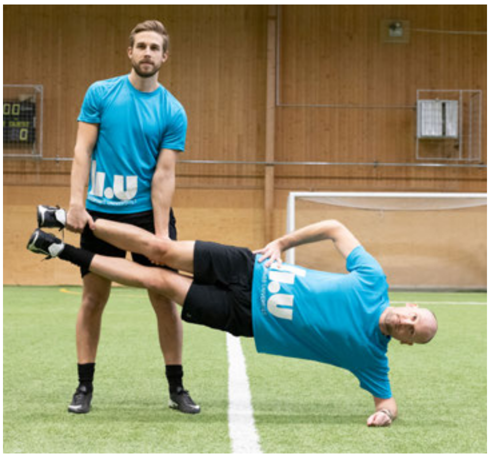

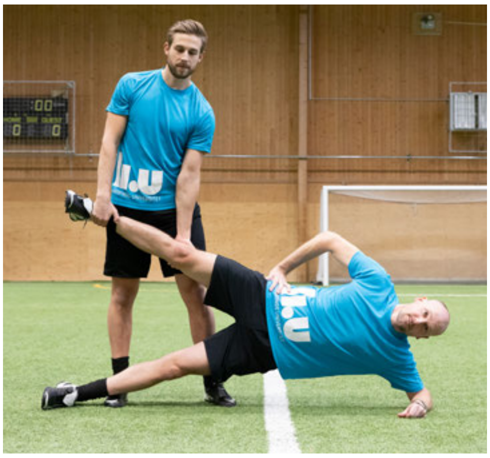

3. Copenhagen adduction – lång hävarm

- Medspelaren stabiliserar ben, tyngden vid fotleden
- Lyft det undre benet samtidigt som du häver kroppen från marken
- Behåll en rak linje genom kroppen
- Arbeta i ett lugnt tempo, 2 sekunder ner, 2 sekunder upp

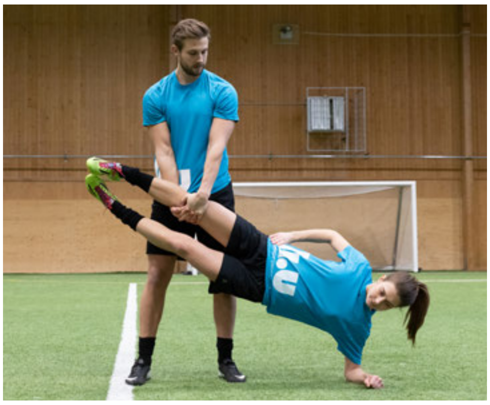

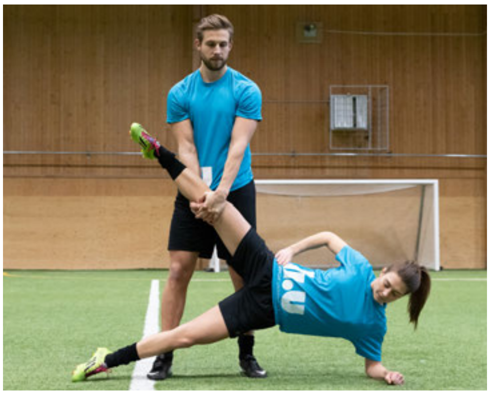

2. Copenhagen adduction – kort hävarm

- Medspelaren stabiliserar ben, tyngden vid knäet
- Lyft det undre benet samtidigt som du häver kroppen från marken
- Behåll en rak linje genom kroppen
- Arbeta i ett lugnt tempo, 2 sekunder ner, 2 sekunder upp

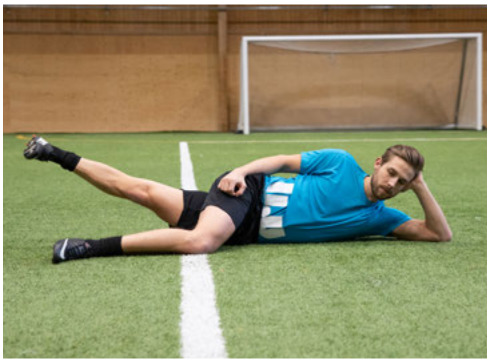

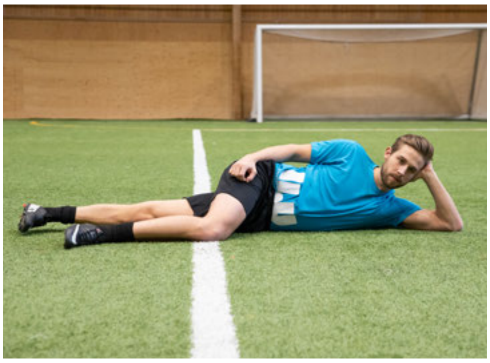

1. Sidliggande adduktion (inåtföring)

- Stabilisera övre benet framför kroppen
- Håll det undre benet sträckt samtidigt som du omväxlande lyfter och sänker benet
- Arbeta i ett lugnt tempo, 2 sekunder upp, 2 sekunder ner

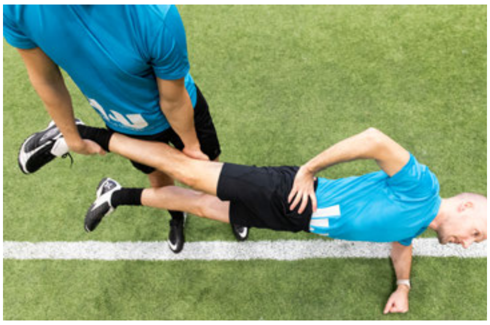

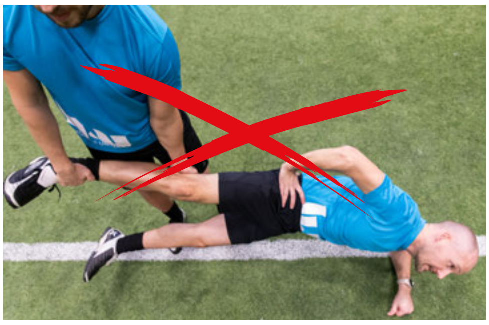

Korrekt utförande

- Exempel på korrekt utförd övning (vänster) respektive felaktigt utförd övning (höger), där kroppen hålls böjd och vrids så att baksida lår aktiveras i stället för insida lår

LINKÖPINGS UNIVERSITET

Komplementövningar

Som alternativ till övningsnivå 2 och 3 (Copenhagen adduction) i originalprogrammet rekommenderar vi dessa 2 övningar om ni önskar begränsa närkontakt mellan spelare. Ni styr användande av övningen med smärtskalan, på samma sätt som i originalprogrammet.

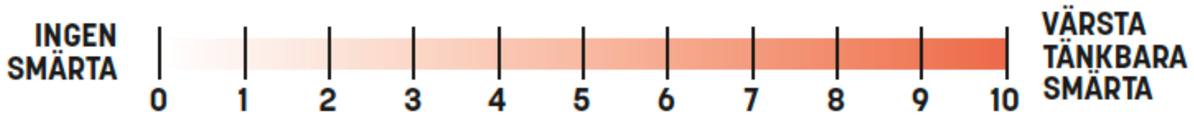

Smärtskala. Minska kraften i övningen om spelaren upplever en smärta >3 i samband med övningen.

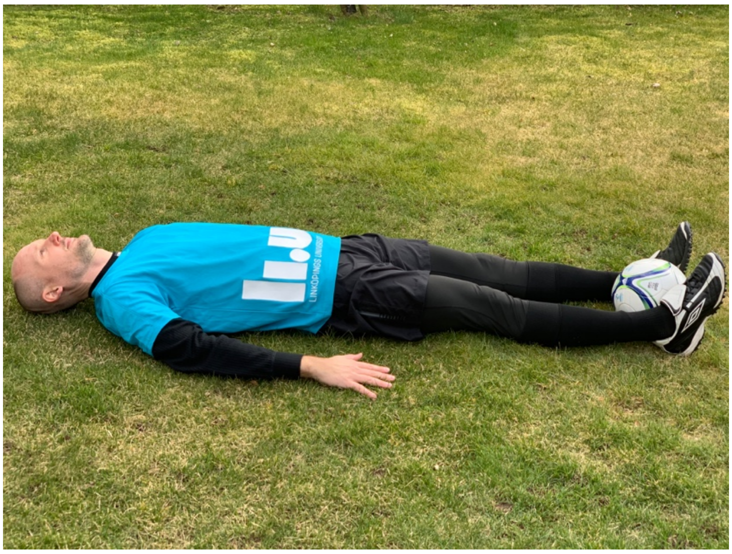

1. Adductor squeeze – raka ben

- Bollen mellan fötterna
- Pressa ihop benen mot bollen med maximal kraft
- Håll 10 sekunder
- Repetera 5 gånger

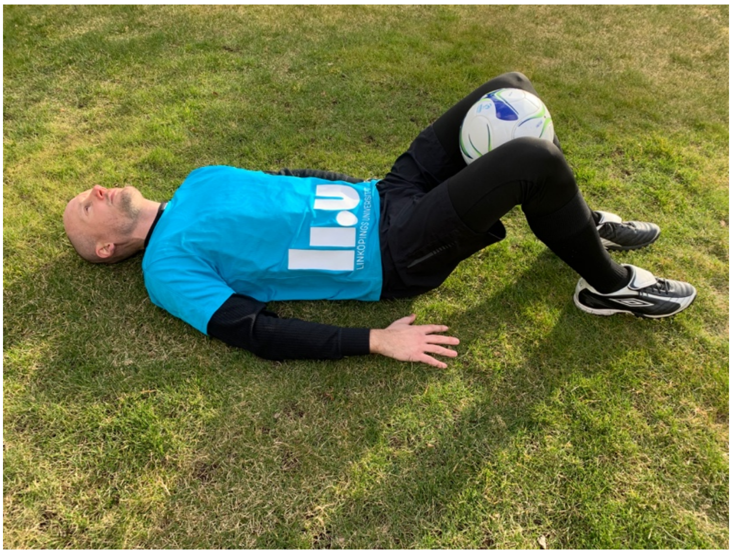

2. Adductor squeeze – böjda ben

- Bollen mellan knäna
- Pressa ihop knäna mot bollen med maximal kraft
- Håll 10 sekunder
- Repetera 5 gånger

LINKÖPINGS UNIVERSITET
