# Supplementary material for: Adherence to Injury Prevention Exercise Programmes in Amateur Adolescent and Adult Football: A Detailed Description of Programme Use from a Randomised Study
Source: Sports Med Open. 2023 Jul 15;9:57. doi: 10.1186/s40798-023-00608-1 (PMC10349794; doi:10.1186/s40798-023-00608-1)
Supplement: Supplementary file 3 — Additional file 3: Table S1. Description of how coaches and players dealt with pain during training. [file 40798_2023_608_MOESM3_ESM.docx]

Sports Medicine – Open. Adherence to injury prevention exercise programmes in amateur adolescent and adult football— A detailed description of programme use from a randomised study. Lindblom H, Waldén M, Hägglund M.

Affiliation for corresponding author: Unit of Physiotherapy, Department of Health, Medicine and Caring Sciences, Linköping University, Linköping, Sweden. Sport Without Injury ProgrammE (SWIPE), Department of Health, Medicine and Caring Sciences, Linköping University, Linköping, Sweden

E-mail address for corresponding author: [hanna.lindblom@liu.se](mailto:hanna.lindblom@liu.se)

Hanna Lindblom, Markus Waldén and Martin Hägglund declare that they have no competing interests.

The study was funded by grants from the Swedish Research Council Ref. no. 2018-03135 and Region Östergötland Ref. No. 922771.

**Additional table 1. Description of how coaches and players dealt with pain during training**

|  | Extended *Knee Control* group | | Adductor group | | Comparison group | |
| --- | --- | --- | --- | --- | --- | --- |
|  | n =20 coaches | n =120 players | n =17 coaches | n =64 players | n =24 coaches | n =105 players |
| Pain during injury prevention exercises, n yes (%) | 6 (30.0) | 13 (10.8) | 5 (29.4) | 15 (23.4) | 3 (12.5) | 21 (20.0) |
| Responses from coaches/players indicating pain during exercises | | | | | | |
| Pain intensity during injury prevention exercises, NRS 0-10, median (IQR)* | N/A | 5.0 (3.5) | N/A | 3.5 (3.0) | N/A | 3.0 (3.5) |
| Changed programme for players with pain, n | 3 | N/A | 5 | N/A | 3 | N/A |
| Carried on with training despite pain, n | 0 | 6 | 0 | 2 | 0 | 8 |
| Alternative exercises when in pain, n | 5 | 2 | 3 | 4 | 2 | 8 |
| Changed to easier exercises, n | 2 | 3 | 4 | 7 | 3 | N/A |
| Changed to fewer repetitions, n | 0 | 2 | 5 | 3 | 1 | 4 |
| Changed the exercise performance, n | 0 | 4 | 1 | 1 | 1 | 8 |
| Gave/received advice about injury prevention, n | 0 | 2 | 1 | 2 | 2 | 2 |
| Changed training set-up, n | 0 | N/A | 1 | N/A | 0 | N/A |
| Removed painful exercises, n | 1 | 3 | 0 | 2 | 0 | 2 |
| Players with pain were excluded from prevention training, n | 2 | 1 | 1 | 6 | 0 | 0 |
| The whole team abstained from IPEP training, n | 0 | N/A | 0 | N/A | 0 | N/A |
| Changed total load for players with pain, n | 2 | N/A | 2 | N/A | 1 | N/A |
| Help/advice from physiotherapist, physician, naprapath, n | 1 | 0 | 1 | 2 | 1 | 4 |

* Based on data from 13 players in extended *Knee Control*, 16 players in adductor group and 21 players in comparison group.

Grey cells with N/A (not applicable) represent questions where this specific group of coaches or players did not receive the question. Abbreviations: IQR–interquartile range, IPEP–Injury Prevention Exercise Programme, N/A–not applicable
